# Altered miRNA Expression Due to Bisphenol A Exposure and Associated Health Implications: A Narrative Review

**DOI:** 10.3390/jox16050159

**Published:** 2026-08-25

**Authors:** Sornali Rani Roy, Soumya Sunil Nair, Aamer Mohammed, Stephen L. Atkin, Edwina Brennan

**Affiliations:** 1 School of Medicine, Royal College of Surgeons in Ireland, Medical University of Bahrain, Busaiteen 15503, Bahrain; sornalir@gmail.com (S.R.R.); 20205745@rcsi.com (S.S.N.); 21200392@rcsi.com (A.M.); 2 School of Postgraduate Studies and Research, Royal College of Surgeons in Ireland, Medical University of Bahrain, Busaiteen 15503, Bahrain; satkin@rcsi.com

**Keywords:** bisphenol A, BPA, endocrine-disrupting chemical, EDCs, epigenetics, micro RNA, miRNA

## Abstract

Bisphenol A (BPA) is a non-persistent industrial chemical widely used in the production of polycarbonate plastics and epoxy resins. Due to its mass production and versatility, BPA is ubiquitous in environmental matrices, leading to human exposure through ingestion, dermal contact, and inhalation. As a known endocrine-disrupting chemical (EDC) with estrogenic activity, BPA exposure has been associated with reproductive, metabolic, immune, oncogenic, and developmental effects. Mechanistically, BPA is reported to exert its toxic effects via multiple pathways, including alterations in epigenetic microRNA (miRNA) expression. miRNAs are endogenous non-coding RNA molecules that regulate gene expression by targeting mRNAs, thereby influencing a wide range of cellular and metabolic pathways involved in development and disease. Importantly, this review consolidates evidence suggesting that the biological effects of BPA may, in part, be mediated through miRNA-driven epigenetic modifications, affecting numerous downstream proteins and signaling pathways. Altered miRNA expression induced by BPA exposure is implicated in diverse health outcomes, including reproductive dysfunction, oncogenesis, metabolic disorders, and neurodevelopmental abnormalities. Notably, BPA exposure predominantly results in the upregulation of specific miRNAs, such as miR-21 and miR-146a, although tissue-specific and sex-dependent variations are evident. In this review, we provide a comprehensive overview of human, in vivo, and in vitro studies investigating BPA-induced miRNA dysregulation and its associated biological effects.

## 1. Introduction

Bisphenol A (BPA; 4,4′-isopropylidenediphenol) is commonly used in the production of plastics due to its hardening capabilities, particularly as a monomer for production of polycarbonate plastics and epoxy resins. Consumer products containing BPA include food and beverage packaging, electronic equipment, medical devices, toys, and paper coatings [1]. With the increased use of plastics, the global production of BPA is forecasted to reach 11.23 million tons by 2029, representing a compound annual growth rate of 6.56% [2]. As a result, BPA is ubiquitous in the environment, with dietary exposure being the primary exposure route, followed by dermal exposure and inhalation [3]. BPA is known to leach from food packaging when residual monomers from manufacturing diffuse or chemically bound monomers undergo hydrolysis [4], with migration rates dependent on factors such as the initial concentration in packaging, food composition, contact time, temperature, repeated use, irradiation, and washing [4,5]. Following oral ingestion, BPA has a terminal half-life of approximately 6 h and is enzymatically conjugated to glucuronic acid in the liver and gut before being excreted in urine [6]. However, this pharmacokinetic profile differs for non-oral routes of exposure. Buccal absorption leads to much higher free BPA internal exposure than gastrointestinal absorption [7,8]. Similarly, dermal exposure lacks the first-pass metabolism, leading to a longer apparent half-life and higher biologically active free BPA [9]. BPA has been detected in urine, serum, blood, breast milk, amniotic fluid, follicular fluid, and saliva [10,11]. According to the European Food Safety Authority, the tolerable daily intake (TDI) of BPA is 0.2 ng BPA/kg body weight. This represents a substantial reduction from previous guidance following evidence of adverse effects on the immune system at much lower doses than previously recognized [12]. Consequently, exposure levels that were once considered acceptable may now exceed the current regulatory limit. Estimated daily intakes across several countries have been reported to be as high as 6.56 µg/kg body weight, far exceeding the current TDI [13].

BPA is a recognized endocrine-disrupting chemical (EDC) due to its estrogenic activity and ability to alter hormone action [14]. BPA exposure has been associated with reproductive effects in males and females, metabolic effects and immune function, liver and kidney impairment, development and migration of certain cancers, and foetal developmental effects [15,16]. The Minderoo-Monaco Commission on Plastics and Human Health estimated that in 2015, health-related costs of plastic-associated chemicals, including BPA, exceeded USD 250 billion globally [17].

The mechanisms by which BPA exhibits its toxic effects include impacts on neuroendocrine function, receptor pathways, enzyme dysregulation, inflammation, and oxidative stress [15]. Gaining greater interest in recent years is the role of epigenetics: altered gene activity in BPA toxicity. Such epigenetic changes include altered expression of microRNAs (miRNAs), which are short, non-coding endogenous RNA transcripts of ~22 nucleotides that repress gene expression post-transcriptionally through complementary binding to target messenger RNA (mRNA) [18]. In addition, miRNAs are reported to mediate intercellular communication given their preferential expression [19] and circulation within extracellular vesicles (EV-miRNA) or binding to proteins [20]. The role of miRNAs is complicated by the fact that a single miRNA may be complementary to hundreds of mRNAs, and a single mRNA can be targeted by many miRNAs [21]. However, miRNAs are important in the control of gene expression, which affects cellular and metabolic pathways that regulate developmental and pathological processes.

Therefore, the aim of this review was to compile and evaluate the body of research on BPA-responsive miRNAs in epidemiological in vivo and in vitro studies while also considering the proposed mechanisms leading to pathophysiological states. With a better understanding of disease onset and etiology, this may, in turn, shed light on the mechanisms underlying environmental disease due to BPA.

## 2. Methods

A comprehensive literature search was conducted to identify relevant studies investigating the association between BPA exposure and altered miRNA expression. The search was performed using three electronic databases: PubMed, Scopus, and Web of Science, covering the period from database inception to June 2026. The search strategy employed a combination of Medical Subject Headings (MeSH) terms and keywords related to BPA exposure and miRNA expression. The following search string was adapted for each database: (“bisphenol A” OR “BPA” OR “4,4′-isopropylidenediphenol” OR “endocrine disrupting chemical” OR “EDC”) AND (“microRNA” OR “miRNA” OR “miR-*” OR “epigenetics” OR “non-coding RNA”).

Studies were considered eligible for inclusion if they met the following criteria: (1) original peer-reviewed research articles (in vivo, in vitro, or human epidemiological studies); (2) investigates BPA exposure and its association with miRNA expression; (3) reports quantitative data on miRNA expression changes; (4) includes appropriate control or comparison groups; and (5) published in the English language. Studies were excluded if they: (1) were review articles, commentaries, conference abstracts, or editorials; (2) lacked original data; (3) did not examine miRNA expression in relation to BPA exposure; (4) were non-peer-reviewed publications or grey literature; (5) were not available in English; or (6) had insufficient data for extraction.

Data from included studies were extracted using a standardized data extraction form. The following information was collected: first author and year of publication, study design (human, in vivo, in vitro), sample characteristics (species, cell/tissue type, sample size), BPA exposure details (dose/concentration, duration, route of exposure), miRNA profiling methodology quantitative real-time PCR (qRT-PCR, microarray, sequencing), differentially expressed miRNAs (upregulated or downregulated), miRNA target genes and validation methods, associated signaling pathways, and key findings related to health outcomes. Our search identified 94 records from PubMed (*n* = 62), Scopus (*n* = 12), and Web of Science (*n* = 20). After manual removal of duplicates, 66 unique records were screened by title and abstract, with 8 studies excluded for not meeting the inclusion criteria. Therefore, a total of 58 studies were included in this review.

## 3. BPA-Induced miRNA Dysregulation and Metabolic Disease

BPA has been implicated in a variety of metabolic dysfunctions, including hypertension, cardiovascular disease [22], and liver damage mediated by oxidative stress [23]. A growing body of evidence also suggests that BPA has obesogenic effects, influencing adipocyte differentiation, increasing adipose tissue accumulation, and disrupting pancreatic β-cell function [24]. Epidemiological studies have additionally linked BPA exposure to abdominal obesity [25]. However, there remain inconsistencies regarding its association with hypertension, dyslipidaemia, and abnormal fasting glucose, highlighting the need for more robust longitudinal studies [25]. Table 1 summarizes key studies exploring the dysregulation of miRNAs by BPA and their potential contributions to metabolic diseases.

### 3.1. Obesity, Insulin Resistance, and Diabetes

In murine preadipocytes, BPA exposure suppressed the expression of miR-21a-5p, a key regulator of adipocyte differentiation [26]. This suppression was associated with increased expression of adipogenic markers PPARγ, C/EBPα, and adiponectin, while also activating glucocorticoid receptor (GR) activity. Additionally, BPA stimulated the p38/MAPK pathway through MKK3 expression. Transfection with miR-21a-5p mimics reversed these effects, demonstrating that the BPA-induced adipogenic phenotype was mediated, in part, through suppression of miR-21a-5p. Mechanistically, miR-21a-5p was shown to specifically target the *map2k3* gene, which encodes MKK3. Consistent with these findings, in vivo studies in Sprague–Dawley rats demonstrated that overexpression of miR-21a-5p attenuated BPA-induced obesity, highlighting the potential therapeutic role of miR-21a-5p in mitigating BPA-related metabolic disruptions [26].

Further investigations in male C57BL/6 mice exposed to high (500 μg/kg/day) and low (50 μg/kg/day) doses of BPA revealed significant disruption of glucose homeostasis, with increased pancreatic insulin secretion linked to insulin resistance [27]. These alterations were associated with changes in the expression of *Pdx1*, a gene critical for insulin production. Interestingly, miRNAs such as miR-338 and miR-200a were differentially regulated in the pancreatic islets, with miR-338 downregulated at high BPA doses and miR-200a downregulated at low doses. In vitro experiments confirmed that miR-338 regulates *Pdx1* expression and contributes to BPA-induced insulin secretory dysfunction. The study proposed that the G-protein-coupled estrogen receptor (GPR30)/glucagon-like peptide 1 receptor (Glp1r)/miR-338-Pdx1 axis might represent a novel pathway through which BPA induces pancreatic dysfunction and insulin resistance.

Verbanck et al. [28] further explored the effects of BPA on adipocyte differentiation using preadipocytes from non-diabetic Caucasian female patients. Their study revealed significant changes in miRNA expression at both low (10 nM) and high (10 μM) doses. MiR-337-3p and miR-5703 were consistently upregulated, while 211 mRNAs were upregulated and 126 were downregulated across both doses. Ingenuity pathway analysis (IPA) identified these mRNAs as being associated with cancer gastrointestinal, hepatic, and reproductive system diseases. Notably, EIF2 signaling was identified as one of the top canonical pathways affected by BPA, with MYCN serving as a key upstream regulator. These findings suggest that BPA exposure disrupts critical cellular pathways, even at environmentally relevant doses, underscoring its potential to contribute to metabolic disease development [28].

In a study by Rahmani et al. [29], male Wistar rats exposed to subchronic BPA doses (406 mg/kg/day) exhibited prediabetic symptoms without significant oxidative damage. Sequencing analysis identified 21 differentially expressed miRNAs, including miR-340-5p, miR-676, and miR-375-3p, with bioinformatic analysis highlighting pathways related to insulin resistance, FOXO signaling, and PI3K-Akt signaling. Additionally, *PPARγ* expression was upregulated, while *Pdx1* expression was downregulated, further supporting the role of BPA in disrupting insulin signaling and contributing to prediabetic conditions.

In a study by Yu et al. [30] further highlighting the complex relationship between EDC and obesity, BPA was shown to disrupt energy balance by altering neuropeptide expression, particularly *AgRP* (agouti-related peptide). The research focused on the role of miRNAs in BPA’s regulation of AgRP, with the hypothesis that miRNAs mediate BPA’s effects on hypothalamic gene expression. The study identified miR-34a-5p as a key regulator of *AgRP* through its control of *KLF4*, a transcription factor. BPA treatment decreased miR-34a-5p levels, which, in turn, upregulated both *KLF4* and *AgRP*. In contrast, miR-501-5p, though predicted to target *AgRP*, unexpectedly increased its expression, suggesting an indirect regulatory pathway. The findings underscore the potential of targeting miRNAs, such as miR-34a-5p, for obesity treatment, with target site blockers (TSBs) representing a promising therapeutic strategy to mitigate BPA-induced dysregulation.

Finally, McIlwraith et al. [31] examined the impact of BPA on hypothalamic cell lines (mHypoA-59) and identified 48 differentially expressed miRNAs. Among these, miR-708-5p showed a substantial increase (28.8-fold), which was associated with the upregulation of its parent gene *Odz4*. This upregulation was mediated by C/EBP homologous protein (*Chop*), a transcription factor induced by endoplasmic reticulum (ER) stress. Overexpression of miR-708-5p led to decreased expression of neuronatin, which is involved in obesity regulation, and increased levels of the appetite-stimulating neuropeptide Y (*Npy*). These findings suggest that BPA exposure may disrupt energy homeostasis, potentially contributing to obesity through alterations in hypothalamic signaling.

Although these findings suggest that BPA-induced miRNA dysregulation contributes to obesity and insulin resistance across multiple tissues, including adipose tissue, pancreatic islets, and the hypothalamus, the evidence is derived mostly from in vitro and animal models, which may not translate directly to human disease.

### 3.2. Liver Function

BPA exposure also has significant effects on liver function, particularly in the context of non-alcoholic fatty liver disease (NAFLD). Lin et al. [32] found that BPA exposure led to significant downregulation of miR-192 in both C57BL/6 mice and HepG2 cells, which was associated with the development of NAFLD and lipid accumulation. Notably, miR-192 directly targeted the 3’ untranslated region (UTR) of *SREBF1*, a pivotal gene in lipogenesis, and its downregulation was linked to altered lipid metabolism. Transfection studies confirmed that overexpression of miR-192 mitigated the effects of BPA, suggesting that miR-192 plays a protective role in regulating lipid metabolism and preventing NAFLD.

Similarly, Vahdati Hassani et al. [33] observed upregulation of miR-122 in the livers of BPA-exposed male Wistar rats. This was accompanied by increased levels of oxidative stress markers (serum 8-isoprostane), liver enzymes (AST, LDH), and altered lipid profiles (HDL, triglycerides, glucose). Furthermore, BPA exposure activated key signaling pathways, including c-Jun N-terminal kinase (JNK), extracellular signal-regulated kinase (ERK1/2), and mitogen-activated protein kinase-activated protein kinase (MAPKAPK), while inhibiting Akt signaling. These findings suggest that miR-122 mediates BPA-induced liver inflammation and necrosis, potentially contributing to liver dysfunction and metabolic disease.

These findings suggest that miR-192 and miR-122 regulate complementary aspects of BPA-induced liver dysfunction. Whereas downregulation of miR-192 promotes lipid accumulation, increased miR-122 expression is associated with enhanced oxidative stress, inflammation, and tissue injury. Both miRNAs have also been proposed as markers of NAFLD and non-alcoholic steatohepatitis [34].

### 3.3. Cardiovascular Disease

The effects of BPA on cardiovascular health have also attracted attention, particularly in relation to blood pressure regulation. In a randomized crossover trial involving 45 non-smoking females aged 60 and above, Kim et al. [35] found that BPA exposure due to canned versus glass-bottled soymilk consumption led to significant changes in miRNA expression. Specifically, miR-30a-5p, miR-580-3p, miR-627-5p, and miR-671-3p were downregulated, while miR-636 and miR-1224-3p were upregulated. MiR-580-3p was associated with changes in systolic blood pressure (SBP), while both miR-671-3p and miR-1224-3p were linked to both SBP and diastolic blood pressure (DBP). The study identified 219 BP-related genes targeted by these miRNAs, with 34 genes strongly associated with hypertension pathways. These findings suggest that BPA exposure may affect blood pressure regulation through miRNA-mediated changes in gene expression, potentially contributing to cardiovascular disease.

The impact of BPA exposure on organ development, particularly on cardiac function, was explored by Rasdi et al. [36] in a study using pregnant Sprague–Dawley rats. Microarray analysis of fetal heart tissue revealed that exposure to BPA resulted in significant upregulation of miR-17-5p, miR-208a-3p, and miR-210-3p. In contrast, only miR-499-5p was significantly upregulated in maternal heart tissue. These changes in miRNA expression were associated with histological alterations in both maternal and fetal heart tissue, including fibrosis, reduced expression of cardiac troponin I (cTnI), and muscle remnants. The study suggests that BPA exposure can disrupt cardiac development and contribute to long-term cardiovascular dysfunction in both the mother and offspring.

**Table 1:** BPA-induced miRNA dysregulation and metabolic disease.

BPA Level/Dose	Study Design; Sample (*n*)	miRNA	miRNA Expression	Methodology	miRNA Target Analysis	Signaling Pathways; Target Genes	Ref.
9 days × 80 μM	In vitro; Murine preadipocytes (3 T3-L1) (*n* = 3)	miR-21a-5p	↓	qRT-PCR	qRT-PCR, Western blotting, luciferase assay	MKK3/p38/MAPK; *map2k3*	[26]
8 weeks × 50, 500 μg/kg/day 2–24 h × 20, 200 nM	Male C57BL/6 mice; pancreatic islets (*n* = 5) In vitro; primary mouse islet (*n* = 3)	miR-338 miR-200a miR-21	↓ ↓ ↑	qRT-PCR	qRT-PCR, Western blotting, luciferase reporter assay, bioinformatic analysis	Glp1r-miR-338-Pdx1; *Pdx1*, *Gpr30*, *Glp1r*	[27]
10 days × 10 nM, 10 μM	In vitro; human preadipocytes (*n* = 3)	miR-337-3p miR-5703 +37 (16 LD) +33 (13 LD)	↑ ↑ ↑ ↓	Microarray	Microarray, bioinformatic analysis, IPA, gene ontology (GO)	EIF2, NAD biosynthesis III, aspartate degradation II, sorbitol degradation I, netrin s, axonal guidance s, remodeling of epithelial, adherens junctions, molecular mechanisms of cancer, sertoli cell–sertoli cell (SC) junctions, and acute phase response; *MYCN*, *FAAH*, *TP53*, *β-E2*, *VEGF*, *TGFB1*, *TNF*	[28]
28 days × 406 mg/kg/day	Wistar male rats; Islets of Langerhans (*n* = 3)	miR-1839-5p miR-340-5p miR-193b-3p miR-676 miR-181a-5p miR-30b-5p let-7c-2-3p miR-375-3p let-7a-1-3p miR-26a-5p miR-126a-3p miR-99b-5p miR-542-5p miR-125b-5p miR-455-3p miR-15b-5p miR-140-5p miR-221-3p miR-1b miR-7b miR-434-5p	↑ ↓ ↑ ↑ ↑ ↓ ↑ ↑ ↑ ↓ ↑ ↓ ↑ ↑ ↑ ↑ ↑ ↑ ↑ ↑ ↑	Next-generation sequencing, qRT-PCR	Bioinformatic analysis, TargetScan, miRWalk, Enrichr, DIANA Tools, GO	Insulin, FOXO, estrogen, PI3K-Akt, leptin, IL-1, IL-6, p38 MAPK, PPAR, TNF-α, IL17, NF-κB, and type 2 diabetes mellitus; *PPARγ*, *Pdx1*, *GAPDH*, *MAPK8*, *mTOR*, *RPS6KA3*, *KIF3A*	[29]
4, 8, 24 h × 100 μM	In vitro; hypothalamic neuronal cells from 8-week-old male mice, mHypoE-41, mHypoE-46 (*n* = 3–6)	miR-501-5p miR-34a-5p miR-34c-5p	↑ ↓ ↓	qRT-PCR	qRT-PCR, bioinformatic analysis, TargetScanMouse, DIANA TarBase	;Krüppel-like factor 4 (*KLF4*), agouti-related peptide (*AgRP*)	[30]
4 and 24 h × 100 μM	In vitro; hypothalamic neurons from adult female mHypoA-59 or embryonic male mHypoE-46 mice clonal cell lines (*n* = 3–4)	miR-708-5p +18 +30	↑ ↑ ↓	Microarray, qRT-PCR	qRT-PCR, literature review	Calcium; *Odz4*, *Npy*, *Nnat*, *Chop*	[31]
90 days × 50 μg/kg/day 48 h × 0, 0.02, 0.2, and 2 μM	C57BL/6 male mice; liver cells (*n* = 4) In vitro; human HepG2 cells (*n* = 3)	miR-192 miR-33 miR-34a miR-122	↓ ↓ (in vivo) ↑ (in vitro) ↑ (in vitro)	qRT-PCR	qRT-PCR, Western blotting, luciferase reporter assay	Lipogenesis and AKT; *SREBF1*, *Fasn*, *Cd36*, *Acacb*, *Scd1*, *Pparg*, *Gpam*, *LDLR*, *APOB*, *APOOC3*, *MTTP*, *DGAT1*	[32]
20 days × 0.5 mg/kg/day	Male Wistar albino rats; liver tissue (*n* = 4–6)	miR-122	↑	qRT-PCR	Western blotting	Akt, JNK, ERK1/2, and MAPKAPK;	[33]
Canned vs. glass-bottled soymilk consumption	Randomized, crossover intervention trial non-smoking females; blood (*n* = 45)	miR-30a-5p miR-580-3p miR-627-5p miR-671-3p miR-636 miR-1224-3p	↓ ↓ ↓ ↓ ↑ ↑	NanoString technology	Bioinformatic analysis, DIANAmT, miRanda, miRDB, miRWalk, RNAhybrid, PICTAR4, PICTAR5, PITA, RNA22, and TargetScan; Database for Annotation, Visualization and Integrated Discovery (DAVID), Cytospace V3.4.0, IPA	Cardiovascular, metabolic, neurological, cancer, immune, psych, developmental, and renal diseases; *ADRA2A*, *SCN1A*, *ST8SIA4*, *STK39*, *BMPR2*, *PPARGC1A*, *CREB5*, *COMT*, *SERPINE1*, *LPL*, *APOB*, *BDKRB2*, *ATP1A2*, *AGT*, *PON1*, *ADRA1B*, *LEP*	[35]
20 days × 5, 20 ppm	Sprague–Dawley rats; maternal and fetal heart tissue (*n* = 5)	Fetal miR-17-5p miR-208a-3p miR-210-3p Maternal miR-499-5p	↑ ↓↑ ↑ ↑	Microarray	N/P	Cardiac disease	[36]
24 h × 10 μM	In vitro; human periodontal ligament stem cells (hPDLSCs) differentiated into endothelial cells (e-hPDLSCs) (*n* = N/S)	miR-1233-5p miR-193b-5p miR-26a-5p miR-6084 miR-6124 miR-6165 miR-619-5p miR-6778-5p miR-6880-5p miR-8075 miR-1343-5p miR-17-5p miR-4270 miR-4298 miR-4441 miR-4673 miR-5196-5p miR-6127 miR-6133 miR-6748-5p miR-6754-5p miR-6756-5p miR-6875-5p miR-939-5p miR-221-3p miR-222-3p miR-551b-5p miR-6511b-5p miR-664b-5p miR-665 miR-6762-5p miR-6798-5p miR-6803-5p miR-6827-5p miR-6845-5p miR-2467-3p miR-4322 miR-6796-5p	↓ ↓ ↓ ↓ ↓ ↓ ↓ ↓ ↓ ↓ ↓ ↓ ↓ ↓ ↓ ↓ ↓ ↓ ↓ ↓ ↓ ↓ ↓ ↓ ↓ ↓ ↓ ↓ ↓ ↓ ↓ ↓ ↓ ↓ ↓ ↓ ↓ ↓	Microarray	Immunofluorescence, Western blot, bioninformatic analysis, Ingenuity Expert Findings, TarBase, TargetScan, miRecords	Angiogenesis; *PECAM1*, *VEGF*, *VEGFR*, *vWF*	[37]

(N/S), not specified; ↑, upregulation; ↓, downregulation; (N/P), not performed.

Another study examined angiogenesis mediated by BPA (10 μM) in endothelial differentiated human periodontal ligament stem cells (e-hPDLSCs) [37]. The angiogenesis markers, PECAM1, VEGF, VEGFR, and vWF, were significantly more highly expressed in BPA-treated e-hPDLSCs, while undifferentiated hPDLSCs had reduced expression. Microarray analysis of EVs from e-hPDLSCs-treated cells identified 395 dysregulated miRNAs. Using bioinformatic analysis, the authors report 10 miRNAs that targeted *PECAM1* (miR-1233-5p, miR-193b-5p, miR-26a-5p, miR-6084, miR-6124, miR-6165, miR-619-5p, miR-6778-5p, miR-6880-5p, and miR-8075), 14 miRNAs that targeted *VEGF* (miR-1343-5p, miR-17-5p, miR-4270, miR-4298, miR-4441, miR-4673, miR-5196-5p, miR-6127, miR-6133, miR-6748-5p, miR-6754-5p, miR-6756-5p, miR-6875-5p, and miR-939-5p), 11 miRNAs that targeted *VEGFR* (miR-221-3p, miR-222-3p, miR-551b-5p, miR-6511b-5p, miR-664b-5p, miR-665, miR-6762-5p, miR-6798-5p, miR-6803-5p, miR-6827-5p, and miR-6845-5p) and 3 miRNAs targeting *vWF* (miR-2467-3p, miR-4322, and miR-6796-5p. The authors suggest that miRNA suppression resulted in upregulated PECAM-1, VEGF, VEGFR, and vWF protein expression, driving uncontrolled angiogenesis through epigenetic dysregulation of extracellular vesicle cargo. However, further mechanistic studies are required to verify this claim.

**Figure 1:**
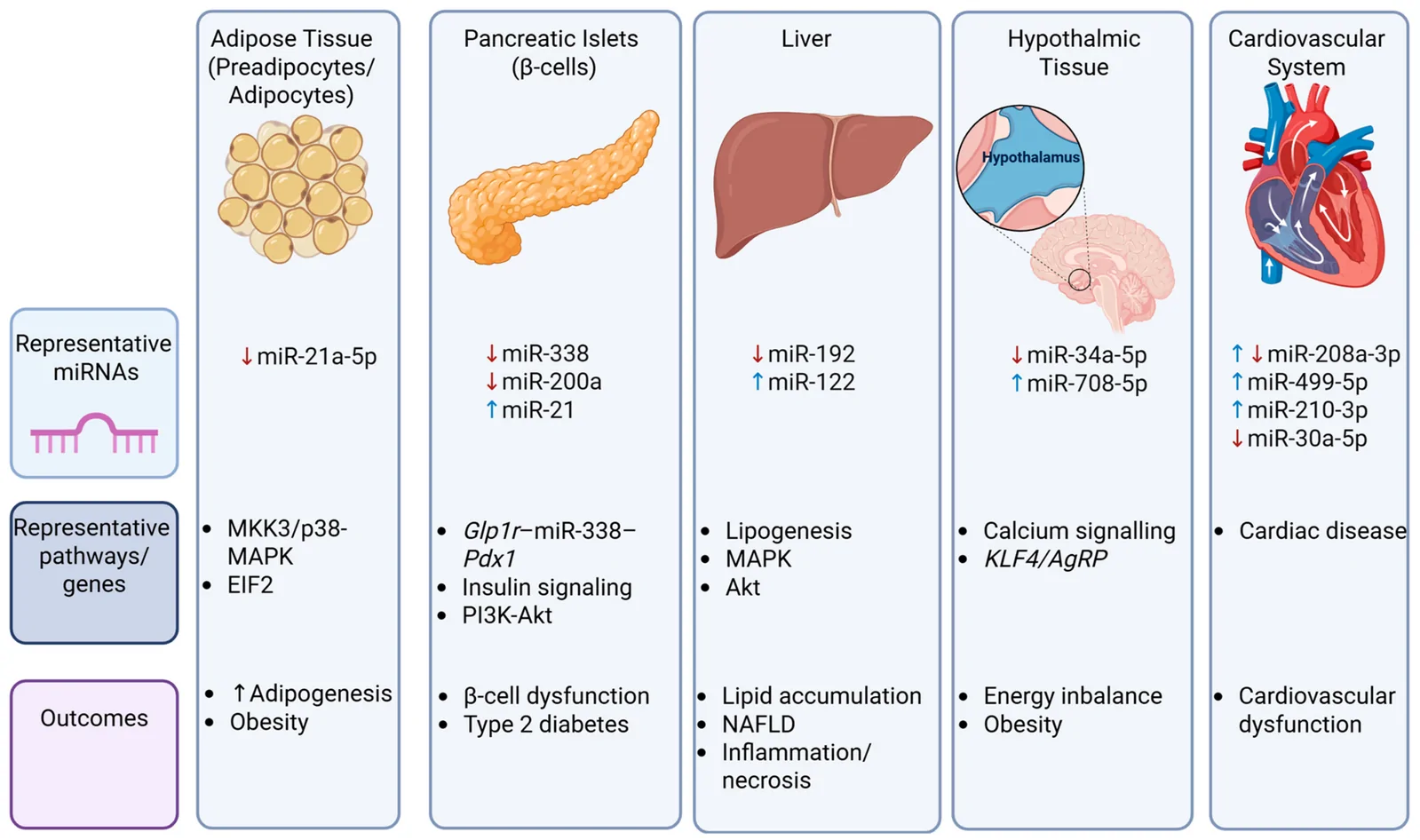
Summary of principal tissues affected by BPA-induced miRNA dysregulation in metabolic disease. Red arrow, downregulated; blue arrow, upregulated; miR, microRNA; MKK3/p38-MAPK, mitogen-activated protein kinase 3 p38 mitogen-activated protein kinase; EIF2, eukaryotic initiation factor 2; *Glp1r*-miR-338-*Pdx1*, glucagon-like peptide 1 receptor miR338 pancreatic and duodenal homobox; PI3K-Akt, phosphoinositide 3-kinase protein kinase B; MAPK, mitogen-activated protein kinase; Akt, protein kinase B; NAFLD, non-alcoholic fatty liver disease; *KLF4/AgRP*, krupple-like factor 4 agouti-related peptide. The illustration was created 15 July 2026 using BioRender.com (with publication license).

A schematic summary of the principal metabolic tissues affected by BPA-induced miRNA dysregulation is presented in Figure 1.

## 4. BPA-Induced miRNA Dysregulation and Cancer

The potential carcinogenicity of BPA remains a topic of ongoing debate. While some studies suggest that BPA may contribute to cancer development through known molecular mechanisms [38], other studies have found insufficient evidence linking BPA to cancer in both animal models and human epidemiological studies [39]. Despite BPA not being classified as a human carcinogen by regulatory authorities, its potential role in cancer remains an area of active investigation. Table 2 summarizes key studies that investigate how BPA may disrupt miRNA regulation and its potential implications for cancer development.

### 4.1. Liver Cancer

Studies have suggested that BPA exposure may contribute to liver carcinogenesis, particularly through the dysregulation of miRNAs. Meng et al. [40] investigated miR-21 expression in human hepatocarcinoma BEL-7402 cells and found that miR-21 levels increased at lower BPA concentrations (100 mM to 1 nM). Given that miR-21 is frequently upregulated in various cancers [41], these findings raise concerns about the potential carcinogenic effects of BPA at environmentally relevant doses.

In a similar way, Kim et al. [42] examined the effects of BPA (68 μM) on human hepatoma HepG2 cells and found increased expression of several miRNAs, including miR-22, miR-1300, miR-941, miR-338-5p, miR-572, miR-671-5p, and miR-595. Of these, miR-22 was significantly upregulated and identified as targeting genes involved in apoptosis and MAPK signaling, such as *CASP7*, *NET1*, *MAPK1*, and *MAPK3*. Inhibition of miR-22 led to the increased expression of these genes, suggesting that miR-22 plays a critical role in regulating cellular pathways that may contribute to liver carcinogenesis.

Renaud et al. [43] explored the impact of chronic BPA exposure on miRNA and mRNA expression in the adult liver of zebrafish, which serves as a model for studying human cancer. Using high-throughput sequencing, the study identified 14 miRNAs that were significantly upregulated and one miRNA, miR-2189, that was downregulated in response to BPA exposure. Further analysis revealed that 6188 target mRNAs were deregulated, with 1491 exhibiting the expected upregulation or downregulation based on changes in miRNA expression. Notably, human orthologs of these genes were enriched in pathways related to NAFLD, oxidative phosphorylation, mitochondrial function, insulin signaling, and adherens junctions. Heatmap analysis also revealed that genes associated with cell cycle regulation, apoptosis, and autophagy were generally upregulated, while genes involved in NAFLD and oxidative phosphorylation were downregulated. These findings underscore the significant impact of BPA exposure on a wide array of biological pathways, suggesting a potential role for BPA in liver carcinogenesis by disrupting normal cellular processes [43].

### 4.2. Colon Cancer

In the context of colon cancer, BPA exposure has also been shown to alter miRNA expression, affecting tumorigenic pathways. Oldenburg et al. [44] exposed Caco-2 cells, a human colon cancer cell line, to varying concentrations of BPA (0.001 to 10 μg/mL) to model gastrointestinal exposure. They found that exposure at 0.1 and 1 μg/mL BPA resulted in a significant upregulation of miR-146a-5p. Bioinformatic analysis indicated that the cancer-related cell signaling pathways were top-ranked, with key target genes such as *TGFB1*, *MYC*, *ErbB*, *BRCA1*, and *EGFR* implicated in processes related to cell growth, invasion, and apoptosis. These results suggest that BPA’s effects on miR-146a-5p expression could contribute to the disruption of normal cellular processes, potentially promoting cancer progression in the colon.

Similarly, Lozano-Herrera et al. [45] examined the effects of BPA on HT-29 colon cancer cells, focusing on the p53 tumor suppressor pathway. Their analysis revealed significant changes in the expression of genes involved in this critical pathway, with upregulation of *CASP2* and *ESR1* and downregulation of genes such as *ATR*, *BBC3*, *MLH1*, *PTEN*, *RB1*, *SIRT1*, *STAT1*, *TADA3*, and *TP53PP2*. Further investigation identified miR-200c and miR-141 as key miRNAs involved in the inhibition of apoptosis and promotion of metastasis as they directly targeted *PTEN* and downregulated its expression. These findings suggest that BPA exposure promotes colon cancer progression by altering key miRNAs and their downstream targets, disrupting apoptosis, and enhancing cell cycle progression.

### 4.3. Nasopharyngeal and Lung Cancer

In addition to colon cancer, BPA has been implicated in the development of nasopharyngeal and lung cancers through similar mechanisms. Zeng [46] exposed human nasopharyngeal carcinoma (NPC) cells (CNE2, CNE1, and 5-8F) to 10 nM BPA and found that exposure triggered malignancy via the Wnt/β-catenin pathway. Transfection studies showed that BPA-induced activation of β-catenin was mediated by increased expression of miR-214-3p, which stabilized *CTNNB1* mRNA and prevented β-catenin phosphorylation. These changes suggest that BPA-induced alterations in miRNA expression can activate oncogenic pathways, contributing to NPC malignancy.

The effects of BPA on lung cancer were also investigated by Oldenburg, Fürhacker, Hartmann, Steinbichl, Banaderakhshan, and Haslberger [44] (Section 4.2), who exposed human lung fibroblasts (HLFs) to BPA (0.001–10 μg/mL). The study revealed upregulation of miRNAs such as miR-24-3p, miR-21-5p, miR-146a-5p, and miR-155-5p at 1 μg/mL BPA. These miRNAs were linked to cancer-related signaling pathways, including those involved in cell cycle regulation, apoptosis, focal adhesion, and immune system signaling. Several target genes, including *TGFB1*, *MYC*, *ErbB*, *BRCA1*, and *EGFR*, were identified as key players in cell growth, invasion, and apoptosis. These findings suggest that BPA exposure may contribute to lung cancer by modulating miRNA expression and disrupting critical tumor suppressor and oncogenic pathways.

### 4.4. Breast Cancer

The role of BPA in breast cancer has also been extensively studied, particularly its impact on miRNA expression. Tilghman et al. [47] examined the effects of BPA exposure (10 μM) on miRNA expression in MCF-7 cells, a human breast cancer cell line with estrogen receptor (ER α) α+ and ER α- subtypes. Their findings showed that BPA exposure induced estrogen receptor α (Erα) expression at the transcriptional level and altered the expression of estrogen-responsive genes such as *BCL2*, *CTSD*, *PGR*, *SERPINB5*, *TFF1*, *JUN*, and *FAS*, while genes such as *GABRP* and *GSN* were downregulated. Additionally, BPA exposure resulted in the upregulation of several miRNAs (e.g., miR-638, -663, -1915) and the downregulation of others (e.g., miR-21, -342-3p, -26b). Notably, miR-21, a well-known estrogen-regulated oncomiR, was found to be differentially expressed in ERα+ and ERα- cells, suggesting distinct estrogen receptor-dependent and independent mechanisms of action. The upregulation of target genes such as *SERPINB5* and *PDCD4*, which are directly regulated by miR-21, further supports the hypothesis that miR-21 plays a crucial role in BPA-induced breast carcinogenesis [47]. While this study reports both ERα-dependent and ERα-independent mechanisms, the potential contribution of ERβ and G-protein-coupled estrogen receptor signaling to BPA-induced miRNA dysregulation was not investigated.

In a separate study, Li et al. [48] exposed MCF-7 cells to 10^−5^ M BPA and observed significant increases in cell proliferation and cell cycle progression. The authors identified miR-19a and miR-19b as key oncogenic miRNAs that were upregulated in response to BPA. These miRNAs induced the downregulation of *PTEN*, leading to enhanced activation of the AKT pathway, a hallmark of cancer cell survival and growth. This suggests that BPA may promote breast cancer progression through the miR-19/PTEN/AKT axis.

Deng et al. [49] also investigated the effects of BPA exposure on MCF-7 cells and found that treatment with BPA (0.1, 1, and 10 μM) led to increased cell proliferation and migration. By analyzing a published gene expression dataset [50], they identified dysregulated genes linked to cell cycle regulation, with *PTTG1* emerging as a key regulator. Further experiments, including transfection studies and tissue microarray analysis, revealed that BPA exposure elevated *PTTG1* expression, which, in turn, promoted MCF-7 cell proliferation. This effect was mediated by the inhibition of miR-381-3p, and the study also found a negative correlation between *PTTG1* and miR-381-3p expression in breast cancer tissue.

In another recent study, Chen et al. [51] demonstrated that BPA exposure (10 μM) led to a significant decrease in miR-26b expression in MCF-7 cells, accompanied by increased *Rab31* expression. This disruption affected lysosomal function, promoting cell proliferation and migration through a miR-26b/Rab31-mediated mechanism. The study suggests that BPA-induced changes in miRNA expression contribute to breast cancer progression through effects on exosome secretion and lysosomal activity.

### 4.5. Prostate Cancer

The effects of BPA on prostate cancer were explored by Wang et al. [52], who investigated the impact of low-dose BPA exposure on prostate glands in adult beagle dogs. BPA exposure caused abnormal growth in the prostate glands and upregulation of serum ratios of oestradiol (E2) to testosterone (T) (E2/T) and prolactin (PRL) to T (PRL/T), indicating hormonal imbalances. Microarray analysis identified several miRNAs, such as miR-199, miR-15a, miR-125b, and miR-1, that were upregulated, while miR-222, miR-99b, miR-208b, and miR-204 were downregulated. These miRNAs were linked to key pathways involved in cellular metabolism, intracellular transport, and organelle function. Notably, targets of the downregulated miR-204, such as *KRAS*, *CDKN1A*, *MAPK1*, *VEGFA*, and *BCL2*, were found to be upregulated at both the mRNA and protein levels, highlighting potential targets for preventing BPA-induced prostate tumorigenesis.

### 4.6. Gynecological Cancers

In the context of gynecological cancers, several studies have linked BPA exposure to dysregulated miRNA expression, which may influence cancer progression. Chou et al. [53] investigated human endometrial cancer (EC) RL95-2 cells exposed to low-to-moderate doses of BPA and found that 82 miRNAs were dysregulated, with 13 implicated in EC progression. Pathway analysis revealed that these miRNAs targeted key genes involved in inflammatory responses, WNT signaling, and DNA damage repair. Notably, miR-149 was downregulated, coinciding with decreased expression of DNA repair genes (*ARF6* and *p53*), suggesting that BPA exposure may interfere with DNA repair mechanisms and promote cancer development. On the other hand, upregulation of miR-107 potentially contributed to cell proliferation by affecting the hedgehog signaling pathway.

In a separate study, Hui et al. [54] examined the effects of BPA on ovarian adenocarcinoma cell lines SKOV3 and A2780. They identified several dysregulated miRNAs, including upregulation of miR-21, miR-221, miR-222, and miR-19a, which are associated with cancer progression. Notably, miR-21 and miR-222 were confirmed to be upregulated by qRT-PCR, with downregulation of their target genes, such as *SERPINB5* and *TIMP3*, suggesting their involvement in tumorigenesis. Additionally, the p53 signaling pathway was found to be impacted, further supporting a role for BPA in promoting ovarian cancer.

Márton et al. [55] investigated the effects of BPA exposure on intracellular and cell-free miRNA expression in human epithelial ovarian cell lines PEO1 (ERα-positive) and A2780 (ERα-negative). The results revealed significant changes in the expression of targeted miRNAs, including miR-200a, miR-200b, miR-200c, miR-141, miR-429, and miR-203a. Notably, miR-200a and miR-200b showed significant downregulation after 24 h of BPA exposure in PEO1 cells, as well as in cell-free lysates. However, no significant changes in miRNA expression were observed in A2780 cells, suggesting that the effects of BPA on these miRNAs are associated with ERα signaling. Additionally, BPA exposure led to the upregulation of estrogen-responsive genes, such as *GREB1*, *DEPTOR*, *CA12*, and *RBBP8*, and the downregulation of *CDH1*, a gene linked to epithelial-to-mesenchymal transition (EMT). In silico analysis of published ChIP-seq data revealed a high-affinity binding site for ERα at the miR-200b-3p/miR-200a-3p/miR-429-3p locus, indicating a potential mechanism through which BPA may regulate these miRNAs. Co-culture experiments with PEO1 and A2780 cells further suggested that BPA-induced changes in miRNA expression could promote cell–cell communication, with increased intracellular levels of miR-200b and miR-200c observed in A2780 cells. This finding points to a potential role for these miRNAs in tumor promotion.

Márton et al. [56] extended their research to explore the global mRNA and miRNA expression profiles in PEO1 cells following exposure to 100 nM BPA. Their analysis revealed upregulation of genes involved in the cell cycle, amino acid metabolism, and estrogen-dependent gene expression, including *MYC*, *EGR1*, *NOLC1*, *MYBL1*, *GREB1*, and *CA12*, while genes related to keratinization, cell junction organization, and cell death, such as *RBBP8NL*, *TGMI*, *and NOTCH3*, were downregulated. BPA exposure also altered the expression of 10 miRNAs (five upregulated and five downregulated), although qPCR validation did not fully confirm the miRNA sequencing results. Further bioinformatic analysis identified a miRNA–protein network involving miR-197-5p, miR-320c, and miR-590-5p, with targets related to biosynthetic processes, cellular differentiation, growth, migration, and EMT, as well as various cancer pathways. These findings further support the role of miRNA dysregulation in the estrogenic effects of BPA.

While there is considerable evidence of BPA-induced miRNA dysregulation with potential implications for cancer development, it is important to note that many of the studies used micromolar BPA concentrations that exceed typical human internal exposure by several orders of magnitude, whereas others used concentrations of one to two orders of magnitude higher (e.g., 100 nM and 0.01 μg/mL). However, several studies employed concentrations within or close to reported human exposure levels (e.g., 10 nM and 0.001 μg/mL). These findings suggest that BPA is capable of altering cancer-associated miRNA networks at environmentally relevant concentrations, although whether these epigenetic changes translate into increased cancer risk in humans requires further study.

**Figure 2:**
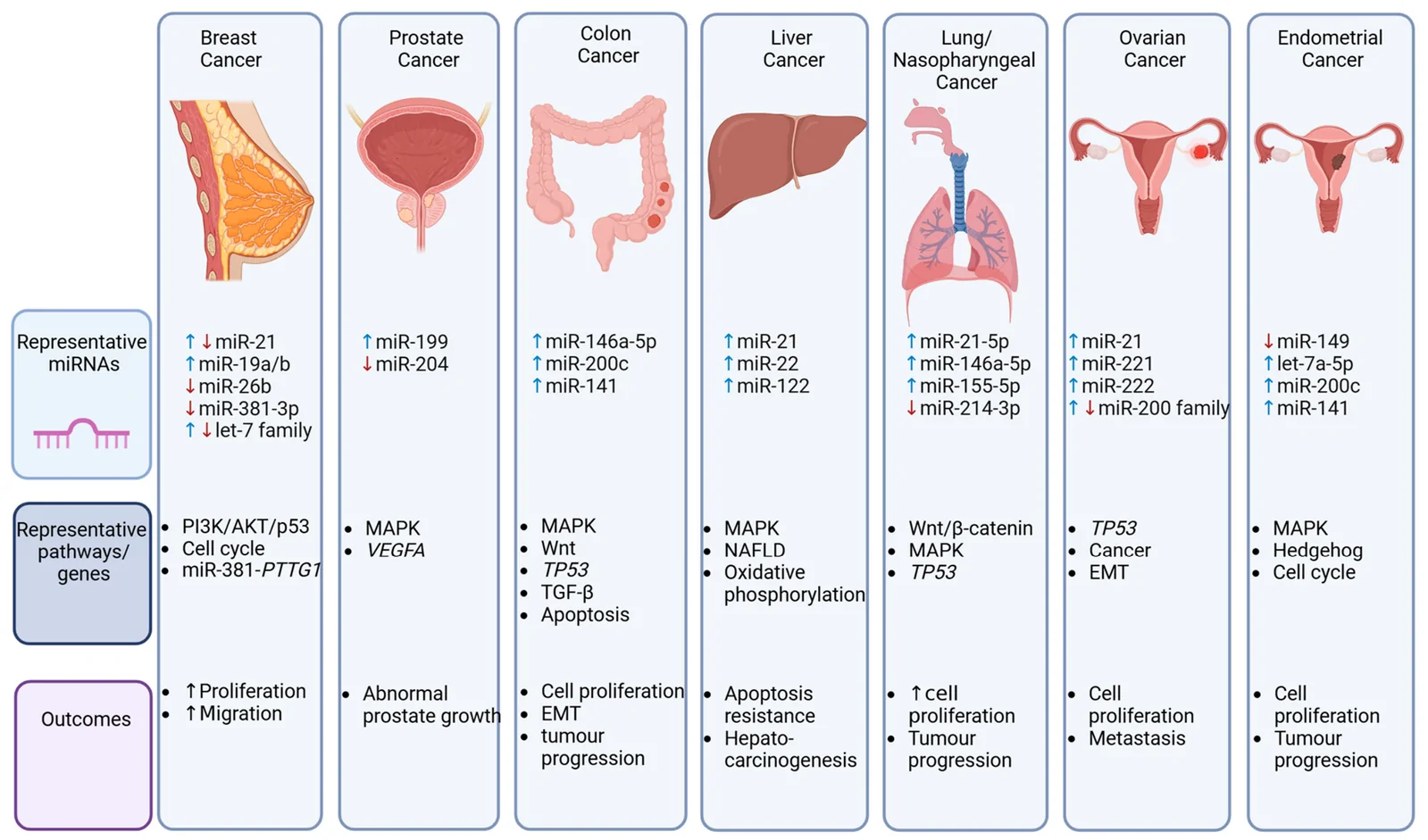
Summary of BPA-induced miRNA dysregulation across major cancer types. Red arrow, downregulated; blue arrow, upregulated; miR, microRNA; PI3K/Akt/p53, phosphoinositide 3-kinase protein kinase B p53; *PTTG1*, pituitary tumor-transforming gene 1; MAPK, mitogen-activated protein kinase; *VEGFA*, vascular endothelial growth factor A; Wnt, wingless + integration-1; TP53, tumor protein p53; TGF-β, transforming growth factor beta; NAFLD, non-alcoholic fatty liver disease; EMT, epithelial–mesenchymal transition. The illustration was created on the 15 July 2026 using BioRender.com (with publication license).

Figure 2 summarizes the major cancer types affected by BPA-induced miRNA dysregulation.

**Table 2:** BPA-induced miRNA dysregulation and cancer.

BPA Level/Dose	Study Design; Sample (*n*)	miRNA	miRNA Expression	Methodology	miRNA Target Analysis	Signaling Pathways; Target Genes	Ref.
3 days × 10^−4^–10^−11^ M	In vitro; human hepatocarcinoma BEL-7402 cells and human mastocarcinoma MCF-7 cells (*n* = 2)	miR-21	↑	qRT-PCR	Literature review	Cancer;	[40]
48 h × 68 μM	In vitro; human hepatoma cells (HepG2 cells) (*n* = 3)	miR-22 miR-1300 miR-941 miR-338-5p miR-572 miR-671-5p miR-595	↑ ↑ ↑ ↑ ↑ ↑ ↑	Microarray, qRT-PCR	Microarray, qRT-PCR, Western blotting, bioinformatic analysis, GO, Kyoto Encyclopedia of Genes and Genomes (KEGG), TargetScan, microCosm	Apoptosis and MAPK; *CASP7*, *NET1*, *SLC7A2*, *MAPK1*, *MAPK3*, *IL1R1*, *ARRB1*, *HSPA1A*	[42]
3 weeks × 100 nM	Male zebrafish; liver tissue (*n* = 2)	miR-430c-3p miR-430b-3p miR-202-5p miR-122 miR-430a-3p miR-499-3p miR-184 miR-499-5p miR-205-5p miR-133a-3p miR-724 miR-458-3p miR-725-3p miR-193a-3p miR-2189	↑ ↑ ↑ ↑ ↑ ↑ ↑ ↑ ↑ ↑ ↑ ↑ ↑ ↑ ↓	High-throughput sequencing	High-throughput sequencing, bioinformatic analysis, miRDeep, Bowti, Gorilla, REViGO, iPathwayGuide, KEGG, miRbase, TARGETSCAN, ToppFun, TargetScanFish, CytoScape	NAFLD, oxidative phosphorylation, metabolic pathways, mitochondrial respiratory electron transport, insulin signaling pathway, adherens junction, and oxidative phosphorylation;	[43]
24 h × 0.001, 0.01,0.1, 1, and 10 µg/mL	In vitro; caco-2 cells and HLFs (*n* = 3)	HLFs miR-24-3p miR-21-5p miR-146a-5p miR-155-5p Caco-2 miR-146a-5p	↑ ↑ ↑ ↑ ↑	qRT-PCR	Bioinformatic analysis, miRNet, KEGG	Cancer, MAPK, focal adhesion, cell cycle, RNA transport, toll-like receptor, Wnt, T-cell receptor p53, apoptosis, neurotrophin, TGF-β, erythroblastic leukemia viral oncogene, and B-cell receptor; *NFAT5*, *APAF1*, *BRCA1*, *E2F2*, *EGFR*, *ICAM1*, *MYC*, *NFKB1*, *OLR1*, *SP1*, *TGFB1*, *VHL*, *RNF11*, *PLEKHA2*, *DCAF10*, *CCND1*, *CDC73*, *ZNF260*	[44]
24 h × 4.4 μM	In vitro; HT-29 colon cancer cell line (*n* = 2)	miR-200c miR-141	↑ ↑	qRT-PCR	mRNA array, qRT-PCR, bioinformatic analysis, GeneGlobe, miRmap	Extrinsic and intrinsic apoptosis and p53; *ATR*, *BBC3*, *MLH1*, *PTEN*, *RB1*, *SIRT1*, *STAT1*, *TADA3*, *TP53PP2*, *CASP2*, *ESR1*, *Erβ*, *GPR30*	[45]
48 h × 10 nM	In vitro; human NPC CNE2, CNE1, and 5-8F cells (N/S)	miR-214-3p	↓	qRT-PCR	qRT-PCR, luciferase reporter assay, Western blotting	Wnt/β-catenin; *CTNNB1*, *CK1α*	[46]
18 h × 10 μM	In vitro; human MCF-7 and -7F breast cancer cells (*n* = 3)	miR-21 miR-342-3p miR-26b miR-27b miR-15b miR-923 let-7f let-7c let-7g miR-638 miR-663 miR-1915 miR-93 miR-320a miR-1308 miR-1275 miR-222 miR-149	↓7↑7F ↓ ↓ ↓ ↓ ↓ ↓ ↓ ↓ ↑ ↑ ↑ ↑ ↑ ↑ ↑ ↑	Microarray, qRT-PCR	Superarray, qRT-PCR, luciferase reporter assay	*SERPINB5*, *PDCD4*, *BCL2*, *CTSD*, *GABRP*, *GSN*, *PGR*, *TFF1*, *JUN*, *FAS*	[47]
4 days × 10^−5^ M	In vitro; human MCF-7 breast cancer cells (*n* = 3)	miR-19a miR-19b	↑ ↑	qRT-PCR	Western blotting	PI3K/AKT/p53; *PTEN*, *AKT*, *MDM2*, *p53*, *PCNA*	[48]
72 h × 0.1, 1, 10 μM	In vitro; human MCF-7 breast cancer cells (*n* = 4)	miR-381-3p	↓	qRT-PCR	Bioinformatic analysis, GO, KEGG, NovelBrain BioCloud, Cytoscape, STRING, mirDIP, qRT-PCR, tissue microarray, luciferase reporter assay, Western blotting	Cell cycle; *PTTG1*	[49]
24 h × 10 μM	In vitro; human MCF-7 breast cancer cells (*n* = 3)	miR-26b	↓	qRT-PCR	qRT-PCR, Western blotting	*Rab 31*	[51]
8 weeks × 2, 6, 18 μg/kg/day	Adult male beagle dogs; prostate gland (*n* = 4)	miR-199 miR-15a miR-125b miR-1 miR-222 miR-99b miR-208b miR-204	↑ ↑ ↑ ↑ ↓ ↓ ↓ ↓	Microarray	qRT-PCR, Western blotting, bioinformatic analysis, GO, KEGG	Cellular metabolic process, intracellular transport, and intracellular organelles; *KRAS*, *CDKN1A*, *MAPK1*, *VEGFA*, *BCL2*, *PTGS2*	[52]
24 h × 10, 10^3^, 10^5^ nM	In vitro; human endometrial carcinoma RL95-2 cells (*n* = 2)	miR-203 miR-205 miR-103a miR-107 miR-200c miR-141 miR-221 let-7a-5p miR-193b miR-423 miR-513 miR-149 miR-765	↑ ↑ ↑ ↑ ↑ ↑ ↑ ↑ ↑ ↑ ↓ ↓ ↓	Microarray, qRT-PCR	Microarray, qRT-PCR, bioinformatic analysis, miRDB, KEGG, Cytoscape	Cancer, hedgehog, cell cycle, adherens junction, and MAPK; *TP53*, *GLI3*, *CCNE2*, *CRK*, *KIF23*, *SAMD2*, *CCDC6*, *FZD3*, *ARF6*, *MAPK9*, *SUFU*, *PRC1*, *MDM2*, *SMAD4*, *DVL1*, *EGLN1*, *JUN*, *MYC*, *LAMC1*, *PRKACA*, *STAT1*	[53]
24 h × 10, 100 nM	In vitro; human ovarian adenocarcinoma SKOV3 and A2780 cells (*n* = 3)	miR-21 miR-221 miR-222 miR-19a miR-7	↑ ↑ ↑ ↑ ↓	qRT-PCR	RNA sequencing, bioinformatic analysis, GO, KEGG, DAVID, GeneMANIA, qRT- PCR	Cancer and p53; *SERPINB5*, *TIMP3*, *MARCKS*, *TSP1*, *IRS-2*	[54]
12, 24 h × 10 nM	In vitro; human epithelial ovarian PEO1 and A2780 cells (*n* = 3)	miR-200a miR-200b miR-200c miR-141 miR-429 miR-203a	↑↓ ↑↓ ↑ ↑ ↑ ↑	qRT-PCR	qRT-PCR, bioinformatic analysis, ChIP-seq analysis	*GREB1*, *DEPTOR*, *CA12*, *RBBP8*, *CDH1*	[55]
8 h × 100 nM	In vitro; human epithelial ovarian PEO1 (*n* = 3)	miR-6795-3p miR-597-5p miR-197-5p miR-5008-5p miR-320c miR-6879-3p miR-3934-5p miR-590-5p miR-636 miR-6806-3p	↓ ↓ ↓ ↓ ↓ ↑ ↑ ↑ ↑ ↑	Sequencing, qRT-PCR	Sequencing, bioinformatic analysis, iDEP.96, Reactome, miRNet, KEGG, qRT-PCR	Regulation of biosynthetic processes, developmental growth, cellular metabolic processes, differentiation, growth, migration, EMT, and cancer; *MYC*, *EGR1*, *NOLC1*, *MYBL1*, *GREB1*, *CA12*, *RBBP8NL*, *TGMI*, *NOTCH3*	[56]

(N/S), not specified; ↑, upregulation; ↓, downregulation; (N/P), not performed.

## 5. BPA-Induced miRNA Dysregulation and Female Reproductive Effects

BPA’s influence extends beyond cancer, with significant evidence indicating its role in disrupting female reproductive health. Exposure to BPA has been linked to various reproductive disorders, including abnormalities in oogenesis, follicle atresia, and polycystic ovary syndrome (PCOS) [57], as well as its contribution to the pathophysiology of endometriosis [58]. In vitro studies have shown that BPA promotes the proliferation, migration, and invasion of human ovarian cancer cells, further suggesting that BPA’s effects on miRNA expression may be pivotal in reproductive system dysfunction [59]. The dysregulation of miRNAs involved in cell cycle regulation and apoptosis may contribute to the observed adverse effects on female reproductive health, highlighting the broader implications of BPA exposure. Table 3 summarizes key studies exploring how BPA may influence miRNA dysregulation and its effects on female reproductive health.

### 5.1. Ovarian Dysfunction

Rodosthenous et al. [60] explored the effects of BPA exposure (20–20,000 ng/mL) on human granulosa cells derived from women undergoing IVF. Using OpenArray to analyze EV miRNA expression and qRT-PCR for cellular miRNA and mRNA profiles, they found that of the 105 EV miRNAs detected, several were differentially expressed. Specifically, Let-7g-5p, miR-191-5p, and miR-532 were upregulated, while miR-212-3p, miR-324-5p, miR-27b-3p, miR-335, and miR-572 were downregulated, although no clear dose–response relationship emerged. Pathway analysis revealed that key targets of miRNAs like Let-7g-5p, miR-212-3p, and miR-27b-3p, including *AKAP8*, *ATP6V1F*, *FADD*, *IGF1*, *MECP2*, and *PPARG*, were overexpressed at high BPA concentrations. These targets are involved in pathways regulating cell cycle progression and the proliferation of gonadal cell lines, which may influence in vitro fertilization (IVF) outcomes. Notably, downregulation of miR-27b-3p was confirmed by qRT-PCR, suggesting that changes in both cellular and extracellular miRNAs may contribute to BPA-induced ovarian toxicity.

Sabry et al. [61] focused on the impact of BPA exposure on miRNA expression in bovine ovaries and oocytes. Using an in vitro bovine model, they examined miRNA expression in matured cumulus–oocyte complexes, oocytes, cumulus cells, and 8-16-cell-stage embryos after BPA exposure at the lowest observed adverse effect level (LOAEL). BPA treatment resulted in increased expression of miR-21, miR-155, and miR-29a, while miR-34c and miR-10b were downregulated, with stage- and cell-specific effects observed across the different ovarian compartments.

The research group later investigated the effects of BPA on miRNA expression in bovine granulosa cells [62]. They found that BPA exposure (0.05 mg/mL) increased miR-21 and decreased miR-10b expression. Knockdown of miR-21 alleviated some of the BPA-induced effects by reducing miR-21 expression and increasing miR-155. Furthermore, BPA exposure led to an increase in *STAT3* and *VMP1*, suggesting that they may act as modulators of miR-21 expression. BPA also increased *PDCD4* and *PTEN* mRNA levels but decreased PDCD4 protein levels, while PTEN protein levels remained unchanged. These findings indicate that BPA may induce apoptotic effects through altered regulation of *PDCD4* via miR-21, although the lack of reversal in miR-21 knockdown experiments suggests that additional pathways may be involved.

The same author group went on to explore DNA methylation under BPA exposure in conjunction with miR-21 knockdown [63]. BPA significantly reduced 5-methylcytosine staining in bovine GCs, which was reversed by miR-21 knockdown, suggesting a mechanism dependent on miR-21. BPA was also shown to increase methylator transcripts *DNMT3A* and *3B* in COCs and *DNMT1* and *DNMT3A* in GCs and demethylator transcripts *TET1*, *2*, *3*, and *TDG* in COCs and *TET 2*, *3*, and *TDG* in GC cells, while *TET1* levels were decreased in GCs independently of miR-21 inhibition. Conversely, at the protein level, BPA decreased DNMT3A and increased TET2 in COCs, whereas in GCs, it decreased both DNMT1 and DNMT3A while increasing TET2. miR-21 inhibition increased the expression of DNMT1 and TET2 protein in COCs and DNMT1 and DNMT3A proteins in GCs. The results suggest an association between miR-21 expression and DNA methylation under BPA exposure, while conflicting mRNA and protein expression may suggest involvement of other miRNAs.

The group further investigated the relationship between BPA exposure, miR-21 downregulation, and connexin (Cx) expression in bovine cumulus–oocyte complexes (COCs) [64]. BPA exposure significantly decreased oocyte maturation rates (*p* = 0.0059), while miR-21 downregulation alone showed a biological but non-significant reduction of 14.5% in polar body extrusion. BPA treatment alone was generally found to increase Cx37, *Cx43*, and *Cx26* expression in COCs and denuded oocytes. However, in cumulus cells, no alteration in Cx expression was observed, but, in tandem with miR-21 knockdown, BPA treatment significantly increased *Cx43* mRNA levels (*p* = 0.0476), suggesting that BPA’s influence on connexin expression may override miR-21 regulatory effects. The study also examined downstream miRNA targets. In COCs, BPA treatment alone generally increased miR-130a, 155, and 378a expression and significantly decreased miR-96 expression. miR-378a, a target of *Cx43* and *Cx37*, increased significantly due to BPA and combination treatment in both denuded oocytes (*p* = 0.0349) and cumulus cells (*p* = 0.0177), while miR-96, a *Cx26* target, displayed significant decreases following BPA exposure in an miR-21-independent manner (*p* < 0.05). These findings demonstrate that BPA disrupts the delicate balance of gap junction proteins through multiple miRNA-mediated pathways, with particular emphasis on the Cx43/miR-378a axis.

Additionally, the group investigated the effects of miR-21 downregulation and exposure to BPA on miRNA expression during bovine oocyte maturation, arrested 8-cell embryos, and blastocysts [65]. Examination of ten key miRNAs involved in oocyte maturation (miR-10b, miR-103a, miR-130a, miR-224, miR-378), embryonic genome activation (miR-155), and pre-implantation development (miR-29a, miR-34c, miR-146a, miR-499) revealed significant dysregulation patterns. In COCs, BPA increased miR-21 and decreased miR-10b, miR-130, miR-224, and miR-499. Co-treatment with BPA and miR-21 knockdown decreased miR-21, miR-130, miR-155, miR-224, and miR-499. In arrested 8-cell embryos, BPA increased miR-10b and decreased miR-29a and miR-34c, whereas co-treatment with BPA and miR-21 knockdown similarly increased miR-10b and decreased miR-29a and miR-34c, with an additional reduction in miR-130. In blastocysts, BPA increased miR-34c, miR-130, and miR-378 and decreased miR-155, whereas co-treatment with BPA and miR-21 knockdown increased miR-103a and decreased miR-155. In addition, BPA treatment alone or co-treatment with miR-21 resulted in significantly reduced maturation cleavage and blastocyst rates, as well as increased blastocyst DNA fragmentation. The results suggest that BPA disrupts stage-specific miRNA regulation during early embryonic development and that BPA and miR-21 may regulate overlapping but distinct regulatory pathways, potentially through miRNA interaction networks.

Collectively, these studies demonstrate that BPA-induced miRNA dysregulation is cell- and stage-specific, influencing multiple regulatory mechanisms leading to impaired female reproductive function, such as cell proliferation, oocyte maturation, gap junction communication, DNA methylation, and early embryonic development. Studies suggest that miR-21 represents a central, although not exclusive, role in mediating BPA toxicity, with additional miRNAs and their interactions also contributing to observed reproductive effects.

### 5.2. Endometrial Dysfunction

Reed et al. [66] studied the impact of BPA exposure on miRNA expression in human endometrial stromal cells. They found that miR-181b, miR-27b, and Let-7c were downregulated in a time-dependent manner following BPA exposure. TargetScan was used to predict potential targets of miR-27b, revealing that its downregulation altered the expression of *VEGFB* and *VEGFC*, genes involved in endometrial vascularization and angiogenesis. This suggests that BPA-induced miR-27b downregulation disrupts the normal processes essential for endometrial function, potentially compromising fertility.

### 5.3. Placental Dysfunction

Several studies have also explored the effects of BPA on miRNA expression in placental cells. Avissar-Whiting et al. [67] conducted microarray analysis on placental cell lines from different stages of development and found significant changes in miRNA expression following BPA exposure. In particular, 4 miRNAs were differentially expressed in 3A cells (first-trimester villous cells) and 14 in HTR-8s (first-trimester extravillous cells), with overlapping upregulation of miR-21, miR-146a, and let-7f/g in both cell lines. Notably, only miR-146a showed a significant effect, with its overexpression leading to decreased cell proliferation and increased sensitivity to bleomycin, indicating its role in BPA toxicity in placental cells.

De Felice et al. [68] performed a comparative analysis of placental miRNA expression in women from a BPA-polluted area and found upregulation of 12 miRNAs in placentas from malformed fetuses, with miR-146a showing strong induction. Pathway analysis linked miR-146a to significant biological processes, including those related to cancer, cardiovascular diseases, and the endocrine system, highlighting its potential role in mediating BPA-induced placental dysfunction.

In contrast, Li et al. [69] observed no correlation between miRNA expression and BPA levels in placental samples from normal-term pregnancies, suggesting that the relationship between BPA exposure and placental miRNA expression may be more complex and context-dependent.

**Figure 3:**
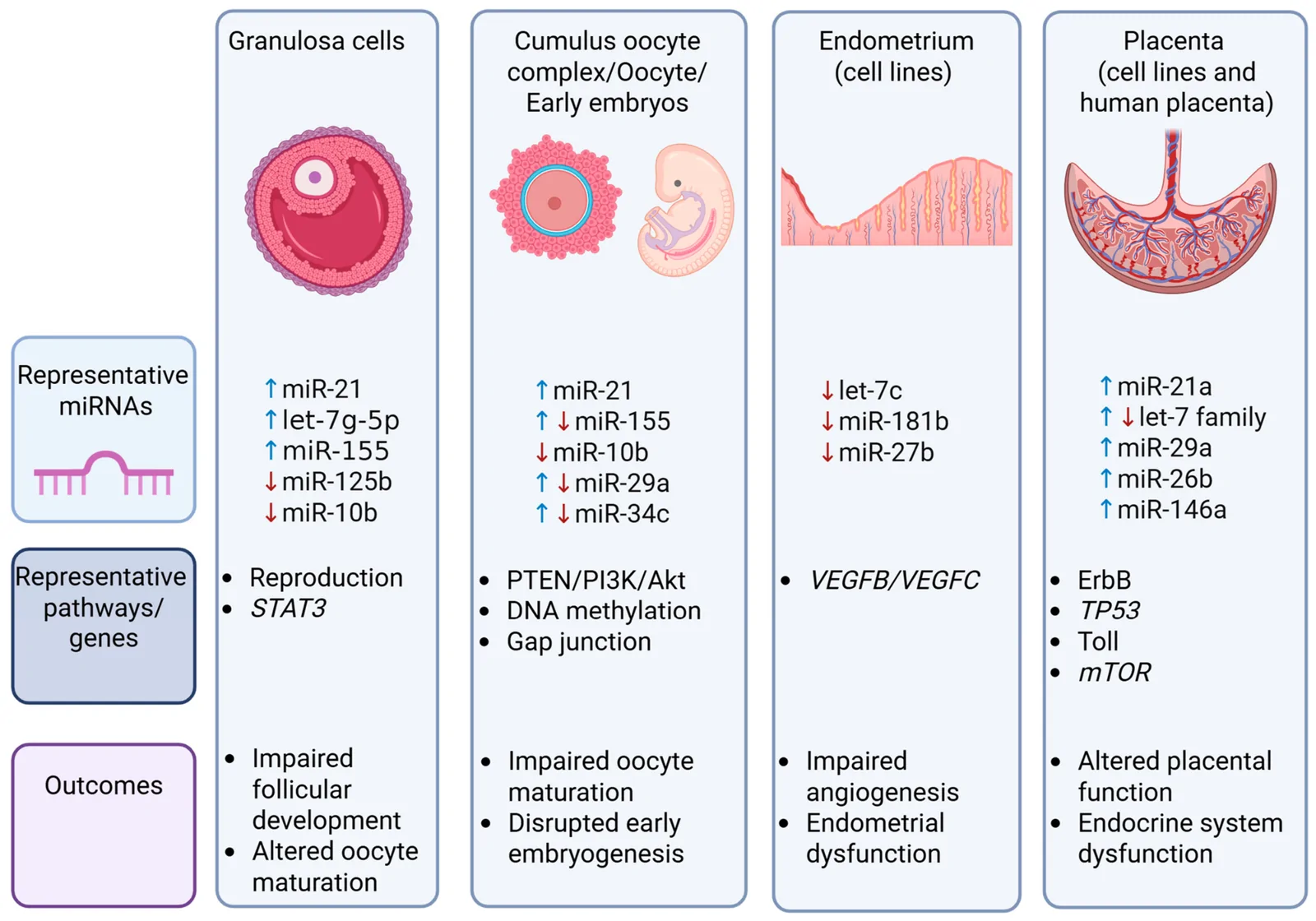
Summary of BPA-induced miRNA dysregulation in female reproductive tissues. Red arrow, downregulated; blue arrow, upregulated; miR, microRNA; *STAT3*, signal transducer and activator of transcription 3; PTEN/PI3K/Akt, phosphatase and tensin homolog phosphoinositide 3-kinase protein kinase B; *VEGFB* and *C*, vascular endothelial growth factor B and C; ErbB, erythroblastic leukaemia viral oncogene homolog; *TP53*, tumor protein p53; Toll, toll receptor; mTOR, mechanistic target of rapamycin. The illustration was created on the 15 July 2026 using BioRender.com (with publication license).

An overview of the principal female reproductive tissues affected by BPA-induced miRNA dysregulation is provided in Figure 3.

## 6. BPA-Induced miRNA Dysregulation and Male Reproductive Effects

BPA has also been shown to negatively impact male reproductive health. Studies in rodent models have demonstrated that BPA exposure leads to decreased sperm concentration and motility [16]. In humans, BPA exposure has been linked to altered hormonal levels and semen characteristics, including reduced sperm concentration and total sperm count. A systematic review and meta-analysis by Lü et al. [70] revealed that urinary BPA levels negatively correlated with sperm concentration and total sperm count, while also being associated with higher levels of sex hormone-binding globulin (SHBG), E2, and lower biologically active androgen levels. These hormonal changes suggest that BPA exposure may have significant repercussions for male fertility, potentially impairing reproductive health. Table 4 outlines key studies examining the impact of BPA on miRNA dysregulation and its effects on male reproductive health.

### 6.1. Cell Function and Tissue Homeostasis

In their study of the effects of BPA exposure on reproductive cell function, Cho et al. [71] investigated miRNA expression in mouse Sertoli testicular cells (TM4) after 3 and 24 h of BPA exposure (20 μg/mL). The microarray analysis revealed 52 and 78 miRNAs with at least a two-fold change in expression at 3 and 24 h, respectively. Among these, 37 miRNAs were differentially expressed at both time points, prompting further bioinformatic analysis. Functional categorization of the genes associated with the altered miRNAs showed that those upregulated were related to metabolism, while genes linked to the downregulated miRNAs were primarily involved in the cell cycle and reproductive processes. Notably, higher exposure to BPA led to an increased number of downregulated miRNAs, indicating a disruption in cellular homeostasis.

In a separate study on mouse spermatocyte-like GC-2 cells, Li et al. [72] exposed the cells to 120 μM BPA and found a significant inhibition of DNA replication, which coincided with alterations in both miRNA (689 upregulated, 98 downregulated) and mRNA (2978 upregulated, 2381 downregulated) expression. Among the miRNAs that exhibited a two-fold differential expression, 48 were linked to spermatogenesis. miR-214-3p, miR-335-5p, miR-29a-5p, miR-152-5p, and miR-340-3p were notably downregulated in response to BPA exposure and associated with apoptotic pathways. Functional analysis through transfection of miR-214-3p demonstrated its role in inhibiting GC-2 cell proliferation, with predicted targets such as *Clock*, *Clasp1*, and *Fbxl7*, which are involved in cell fate regulation. Further investigations by Li et al. [73] confirmed the downregulation of several genes, including *Akt1*, *Cd59a*, and *Pkn3*, in response to miR-214-3p overexpression. Notably, *Akt1*, a key kinase in cell survival, was shown to be directly targeted by miR-214-3p, leading to decreased cell viability and contributing to BPA-induced apoptosis.

In male Fischer 344 rats, BPA exposure was linked to reduced levels of E2 and T, impaired erectile function, and an increase in fat accumulation, myofibroblasts, and apoptosis within the corporal tissue [74]. The authors identified several upregulated genes related to inflammation (e.g., interleukins, cytokines) and downregulated genes related to EMT and fibrosis (e.g., keratins). miRNA profiling revealed 25 miRNAs with significant changes in expression, including miR-296-3p, miR-200 (a, b, c), miR-203a, miR-205, and miR-494-3p, which were associated with inflammation, EMT, and fibrosis. These findings further underscore the role of miRNAs in mediating the harmful effects of BPA exposure on male reproductive tissues [74].

### 6.2. Steroidogenesis

Gao et al. [75] explored the effects of BPA on male reproductive function, focusing on miR-146a-5p expression in Leydig cells (LCs) of male C57B/6 mice. They found that BPA exposure led to a dose-dependent increase in miR-146a-5p expression, which correlated with decreased plasma T levels, reduced testis weight, and lower epididymal sperm count. In vivo inhibition of miR-146a-5p partially rescued the BPA-induced disruption of T production, and in vitro experiments using MA-10 cells confirmed that miR-146a-5p inhibition improved progesterone synthesis, which had been suppressed by BPA. Additionally, *Mta3*, a gene involved in the regulation of steroidogenesis, was identified as a potential downstream target of miR-146a-5p. These results suggest that the miR-146a-5p/Mta3 axis plays a significant role in mediating BPA-induced steroidogenic dysfunction in male reproductive tissues.

### 6.3. Infertility

Palak et al. [76] examined miRNA expression in seminal samples from men with varying degrees of infertility. The study found that azoospermia samples contained significantly higher levels of BPA compared to oligoasthenoteratozoospermia and control samples. Using qRT-PCR, they identified four miRNAs—miR-let-7a, miR-let-7b, miR-let-7c (all upregulated), and miR-518f (downregulated)—as being differentially expressed in azoospermia samples. Furthermore, BPA levels positively correlated with miR-let-7a and miR-let-7c expression and negatively correlated with miR-518f. The authors proposed a potential molecular mechanism through the BPA-CYP19-miR-let-7 pathway, based on previous in silico analysis suggesting that the miR-let-7 family targets *Cyp19*. These findings suggest that these miRNAs could serve as biomarkers for BPA exposure and may help explain its role in male infertility.

Additionally, in a study by Santiago et al. [77], the impact of BPA on human sperm miRNA and protein expression was explored in a cohort of 102 Portuguese men. BPA was detected in 88% of seminal plasma samples, though no significant correlation was found with conventional semen parameters. However, small RNA sequencing of 15 normozoospermic samples identified 15 miRNAs significantly correlated with BPA levels involved in embryonic development and stress response. Notably, miR-29b-3p and miR-34b-3p were upregulated in samples with higher BPA concentrations, suggesting that they may be markers for sperm quality and fertility. Proteomic analysis revealed 62 differentially expressed proteins linked to sperm function and early embryo development, with altered levels of DEFB126 (involved in sperm–mucus penetration) and HDAC11 (regulating early embryogenesis).

In a bovine in vitro study, sperm were directly exposed to BPA at 0.05 mg/mL (LOAEL dose) for 4 h, followed by IVF to assess sperm function, embryo development, and miRNA profiles [78]. BPA significantly decreased acrosome reaction levels, reduced cleavage rates, and lowered blastocyst development compared to controls. Blastocysts derived from BPA-treated sperm exhibited fewer total cells and approximately threefold higher DNA fragmentation than controls. qRT-PCR analysis of eight fertility-associated miRNAs in sperm and blastocysts revealed no statistically significant changes, though BPA-exposed blastocysts showed a non-significant trend toward increased miR-130a expression. These findings indicate that BPA impairs sperm function and embryo quality through mechanisms that may operate independently of detectable miRNA dysregulation.

**Figure 4:**
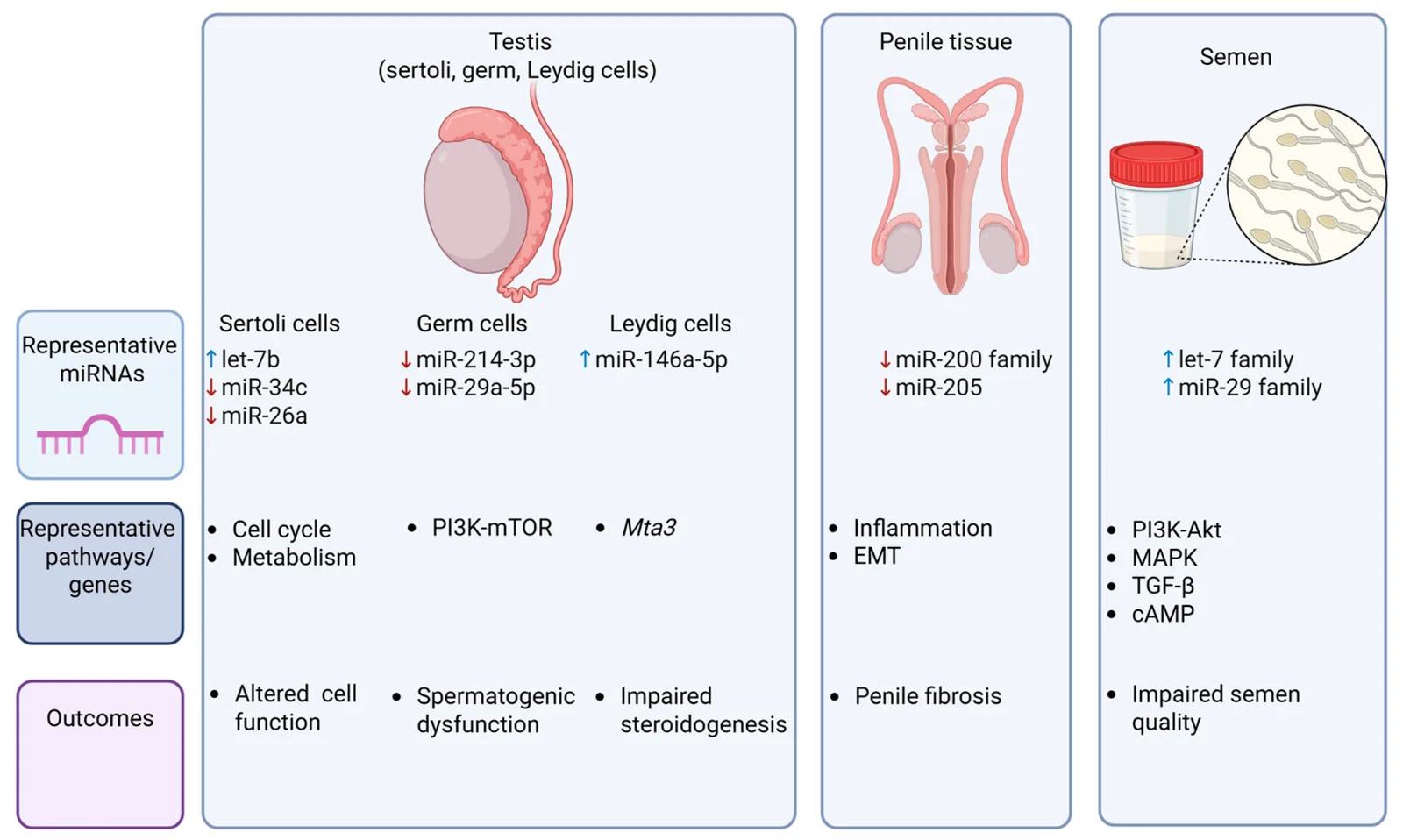
Summary of BPA-induced miRNA dysregulation in male reproductive system. Red arrow, downregulated; blue arrow, upregulated; miR, microRNA; PI3K-mTOR, phosphoinositide 3-kinase mechanistic target of rapamycin; *Mta3*, metastasis associated 1 family member 3; EMT, epithelial–mesenchymal transition; PI3K-Akt, phosphoinositide 3-kinase protein kinase B; MAPK, mitogen-activated protein kinase; TGF-β, transforming growth factor beta; cAMP, cyclin adenosine monophosphate. The illustration was created on the 15 July 2026 using BioRender.com (with publication license).

A summary of miRNA alterations associated with BPA exposure in the male reproductive system is provided in Figure 4.

## 7. BPA-Induced miRNA Dysregulation and Developmental Programming

BPA exposure during pregnancy is increasingly recognized for its potential to disrupt developmental programming in offspring, leading to long-term effects on growth, reproduction, organ development, and neurobehavioral outcomes. Ma, Liu, Wu, Yuan, Wang, Du, Wang, Marwa, Petlulu, Chen, and Zhang [15] reported that prenatal BPA exposure is linked to adverse birth outcomes, including increased risk for cardiovascular disease [79] and obesity [80]. These findings underscore the far-reaching consequences of BPA exposure on both immediate and long-term health. Table 5 outlines key studies examining the impact of BPA on miRNA dysregulation and its effects on developmental programming.

### 7.1. Embryonic Development

In early embryogenesis, BPA exposure can significantly alter gene expression and developmental pathways. Chen et al. [81] investigated the effects of BPA on mouse embryonic stem cells (mESCs) and embryoid bodies (mEBs) in vitro. They observed an increase in the expression of pluripotency markers such as *Oct4, Sox2*, and *Nanog* in both mESCs and mEBs, suggesting that BPA may interfere with normal differentiation processes. Furthermore, BPA exposure led to an upregulation of markers associated with endodermal (*Gata4*, *Sox17*) and mesodermal (*Sma*, *Desmin*) lineages, while markers of the ectodermal lineage (*Nestin*, *Fgf5*) were downregulated. Notably, the expression of miR-134, a known inhibitor of pluripotency, was reduced, indicating that BPA may disrupt the balance of pluripotency and differentiation through altered miRNA expression.

### 7.2. Organ and Bone Development

Puttabyatappa et al. [82] examined the effects of prenatal BPA exposure (0.5 mg/kg/day) on the liver and muscle tissues of female sheep. RNA sequencing revealed that BPA exposure caused dysregulation of 14 miRNAs in the liver, including downregulation of miR-29A, miR-30B, and miR-154A and upregulation of miR-200B, miR-323A, and miR-409. In muscle tissue, miR-26B and miR-29A were downregulated, while miR-181A-1, miR-125B, miR-127, and miR-541 were upregulated. Although no miRNAs were correlated with dysregulated mRNAs, these miRNAs have potential as signatures of prenatal BPA exposure, reflecting its impact on organ development.

Zhu et al. [83] examined rare minnow paternal BPA exposure of 14.03 ± 0.89 μg/L for 14 days on offspring bone development. Exposure resulted in reduced straight-line velocity of paternal sperm and an increased rate of axial dysplasia and changes in craniofacial development in the F1 generation, while the F2 generation was unaffected. MiRNA sequencing of paternal sperm identified 859 miRNAs, of which 13 were upregulated and 15 were downregulated, validated with qRT-PCR. Bioinformatic analysis identified that the miRNAs were associated with protein binding, signal transduction, membrane composition, calcium ion binding, and protein phosphorylation and enriched in Wnt, TGF- beta, oocyte division, endocytosis, cell cycle, adhesion junction, and AMPK signaling pathways. Seven miRNAs were identified to be involved in bone development (PC-3p-133610_29, PC-3p-18101_257, ccr-miR-727–3p, dre-miR-31, oni-miR-7550, PC-3p-2927_1428, and ssa-miR-16b-5p), while four bone development genes were identified (*Runx1*, *Runx2a*, *bmp2a*, and *bmp5*). In the F1 generation, paternal BPA exposure downregulated *bmp2a* and *Runx1* chondrogenic genes, while upregulating aca-miR-16a-5p (which targets bmp2a) at 24 h post-fertilization; however, no changes in ccr-miR-727-3p (targeting Runx1) were detected. The study suggests that paternal BPA exposure causes miRNA disturbances in spermatozoa, which may contribute to alterations in chondrogenic genes, leading to offspring malformation.

### 7.3. Female Reproductive Effects

In a study by [84], pregnant Suffolk ewes were exposed to BPA (0.5 mg/kg) from day 30 to 90 of gestation, and fetal ovaries were collected on days 65 and 90 for analysis. By day 65, the mRNA expression of key steroidogenic enzymes Cyp19 and 5α-reductase was significantly increased compared to controls. However, BPA exposure also led to a reduction in miRNA expression, with 45 miRNAs downregulated on day 65 and 11 miRNAs downregulated on day 90, all showing fold changes greater than 2 compared to controls. Interestingly, only miR-203 was consistently downregulated at both time points and predicted to target ERα and insulin signaling pathways. Despite these predictions, no corresponding changes in mRNA levels of these targets were observed. Additionally, several other miRNAs were predicted to target genes such as Kit ligand and members of the Sry- related high-mobility-group box (*SOX*) family, which are involved in gonadal differentiation and insulin regulation. These results suggest that BPA exposure modifies the expression of key genes involved in ovarian function, gonadal differentiation, and insulin homeostasis, which could have long-term implications for female fertility and health.

Further investigation into the effects of BPA on ovarian function was conducted by Lite et al. [85], who exposed pregnant rats to various BPA concentrations (2.5, 250, 2500 mg/L). By analyzing miRNA expression in ovarian granulosa cells of female offspring, they found that miR-224 was significantly upregulated at high BPA doses. Notably, this upregulation of miR-224 was associated with increased expression of CYP19A1 protein in a dose-dependent manner. Given the observed negative association between BPA exposure and follicle-stimulating hormone (FSH) levels in offspring, as well as the positive association with E2 levels, the authors suggest that upregulation of miR-224 could disrupt normal ovarian function and potentially contribute to pathological conditions later in life, including fertility issues and ovarian disorders.

### 7.4. Male Reproductive Effects

The impact of prenatal BPA exposure on male reproductive health was examined by Ma et al. [86] in Sprague–Dawley rats. The study used microarray analysis to identify abnormal miRNA expression in the testes of fetuses exposed to BPA, finding 564 differentially expressed miRNAs. Among these, miR-361-5p and miR-19b-25p were significantly downregulated at both low (0.05 mg/kg) and high (5 mg/kg) exposure levels, while miR-203a-3p was upregulated at higher BPA doses. Bioinformatic analysis revealed that the target genes of these miRNAs were involved in RNA polymerase II transcription, cancer pathways, and key signaling pathways such as FoxO and VEGF (vascular endothelial growth factor). These altered pathways were associated with observed changes in body weight, LH (luteinizing hormone), T levels, and reproductive system damage in male offspring. The findings suggest that early-life exposure to BPA can lead to dysregulated miRNA expression, which, in turn, disrupts gene function and signaling pathways crucial for male reproductive health and development [86].

### 7.5. Neurodevelopmental Effects

The neurodevelopmental effects of BPA were explored in Long Evans rats by Lichtensteiger et al. [87], who focused on the hippocampus, a region critical for learning and memory. The study found sexual dimorphism in BPA’s effects, with males showing more significant alterations in gene expression than females. In males, genes associated with hippocampal and cerebral cortex development, such as *Gli3*, *Pou2f1*, *Oct1*, *Sox6*, and *Sox11*, were significantly altered. Furthermore, genes involved in growth factor signaling, including *Nrg1*, *Eph4*, *Fzd3*, and *Fgf14*, were also disrupted. Network analysis suggested a potential link between ERα, miR-24-3p, and *Sox6*. In particular, increased expression of ERα was associated with downregulation of miR-24-3p, upregulation of *Sox6*, and downregulation of the Sox6-regulated gene *Pvalb*, which is important in interneuron function. These results suggest that miR-24-3p may play a role in the effects of BPA on learning and memory, potentially via disruption of neuronal signaling pathways.

In a related study by Butler et al. [88] on maternal BPA exposure in California mice (Peromyscus californicus), the authors investigated miRNA expression in the hypothalamus and hippocampus of offspring. At a low dose (5 mg/kg), miR-153 was upregulated in both regions, while miR-181a was downregulated in the hypothalamus and miR-9 was upregulated in the hippocampus, but only in females. Further analysis revealed altered expression of BPA-associated mRNAs: in the hypothalamus, there was an increase in *Avp* (arginine vasopressin), estrogen receptor (*Esr*) 1, *Kiss1* (kisspeptin), and *Lepr* (leptin receptor), while in the hippocampus, *Esr2* was decreased and *Gnrh* (gonadotropin-releasing hormone) was increased. Correlation analysis revealed that miR-9 expression in the hippocampus was positively associated with social interaction, while miR-181a and miR-153 in the hypothalamus were linked to metabolic parameters, such as body fat and lean mass, in a sex-specific manner. These findings suggest that BPA-induced miRNA dysregulation has lasting effects on social behaviors, metabolic regulation, and, possibly, neurodevelopment in both male and female offspring.

Kaur et al. [89] conducted a more comprehensive analysis of global miRNA expression in the same study model, identifying significant changes in miRNA profiles, particularly in females (87 differentially expressed miRNAs at low and 1 at high dose) and males (44 at low and 66 at high dose). Among these, miR-146a, a well-known miRNA involved in immune response and brain function, was upregulated in female offspring exposed to a low dose. Further analysis of its mRNA targets revealed that miR-146a might influence neuronal differentiation and inflammation by modulating genes such as *Cdk5*, *Grid1*, *Klf4*, *Ptpra*, and *Syt14*. Correlation analysis indicated that miR-146a expression was positively associated with social behaviors and negatively correlated with body fat in low-dose females, while in high-dose males, it was linked to body weight and fat percentage. These findings suggest that miR-146a may mediate some of the behavioral and metabolic alterations induced by BPA exposure, highlighting its potential role in neurodevelopmental disorders and metabolic dysfunction.

Further support for the role of miRNAs in BPA-induced neurodevelopmental effects comes from a study by Nayan et al. [90] examining prenatal exposure to BPA in male rats, where exposure at a dose of 5 mg/kg/day led to significant downregulation of miR-19a and miR-539 in the hippocampus. These miRNAs are critical for regulating NMDA receptor subunits (*GRIN2A* and *GRIN2B*), which are key to synaptic plasticity and memory. The downregulation of these miRNAs resulted in a decrease in GluN2A and GluN2B proteins in the hippocampus, leading to significant impairments in both fear and spatial memory by adolescence. This study highlights the crucial role of epigenetic regulation in the developmental impacts of BPA, particularly through disruptions in miRNA expression, which can impair NMDA receptor function and, consequently, cognitive abilities.

### 7.6. Metabolic Effects

In the study conducted by Huang et al. [91] maternal oral BPA exposure at 0.05 mg/kg/day during pregnancy induced obesity in male offspring mice, and maternal injected exosomes from male offspring produced similar impacts in recipient male offspring. Exosomal miRNA sequencing of serum from male offspring identified more than 200 miRNAs. KEGG pathway analysis linked these differentially expressed miRNAs to AMPK, PPAR, and Ras signaling pathways. The most up-regulated miRNAs were miR-124-3p and miR-466i-5p, which were found to be consistently highly expressed in adipose tissue, with levels in liver and serum increasing progressively over time, validated by qRT-PCR. The authors demonstrated in vitro that adipose-derived stem cell exosomes were taken up by hepatocytes, resulting in insulin resistance coinciding with reduced expression of FGF21 and p-AKT. Further sequencing analysis identified 194 DEGs associated with PPAR, insulin, and TNF signaling pathways, with insulin gene expression (*Pparc*, *Fgf21*, *Slc2A4*, *Ppp1r3b*, *Hspa5*, *Nr1d1*, and *Gale*) validated by qRT-PCR. Transfection studies confirmed reduced expression of *PPARγ* and *Fgf21*, mimicked by miR-124-3p overexpression, which was reversed by miR-124-3p knockdown. The study establishes exosomal miRNAs as novel mediators of BPA-induced obesity.

Similarly, BPA exposure at 50 μg/kg/day for 21 weeks induced obesity, IR, and hyperlipidemia in male mice, with these metabolic abnormalities transmitted to adult offspring [92]. In paternal mice, BPA reduced hepatic phosphorylated Akt and *Lepr* expression while increasing sperm miRNA processing proteins Xpo5 and Dicer1. Microarray analysis identified significantly dysregulated miRNAs associated with PI3K-Akt, insulin, IR, AMPK signaling, and non-alcoholic fatty liver disease. In spermatocytes, BPA triggered lipid reprogramming and activated the Srebf1-Pparg axis, driving Dicer1 transcription and elevating multiple sperm miRNAs: miR1a-3p, miR145a5p, miR150-5p, miR486a-3p, miR-700-5p, and miR3068-5p. In the liver of male and female F1 progeny, increased expression of miR149-5p, miR150-5p, and miR700-5p was observed along with decreased expression of *Lepr*, *Igfbp2*, and pAmpk, upregulation of *Srebf1* and *Pparg*, and dysregulation of the Insr–Irs1–Akt cascade. In addition, decreased expression of the cellular senescence marker Cyclin D1 was observed, along with upregulation of key proteins involved in senescence regulation (p53, p16, p21, and p27). miR149-5p, miR150-5p, and miR700-5p were found to directly bind to the 3′UTR of *LepR*, and overexpression in AML12 hepatocytes increased the expression of *Srebf1* and *Scd1* while simultaneously decreasing the expression of *Igfbp2* and *Egfr* and suppressed the phosphorylation of Akt and Ampk. The results suggest that upregulation of sperm miRNAs can be attributed to the influence of BPA on miRNA biogenesis via the Srebf1-Pparg-Dicer1 axis, representing a potential mechanism for the paternal transmission of metabolic abnormalities.

**Figure 5:**
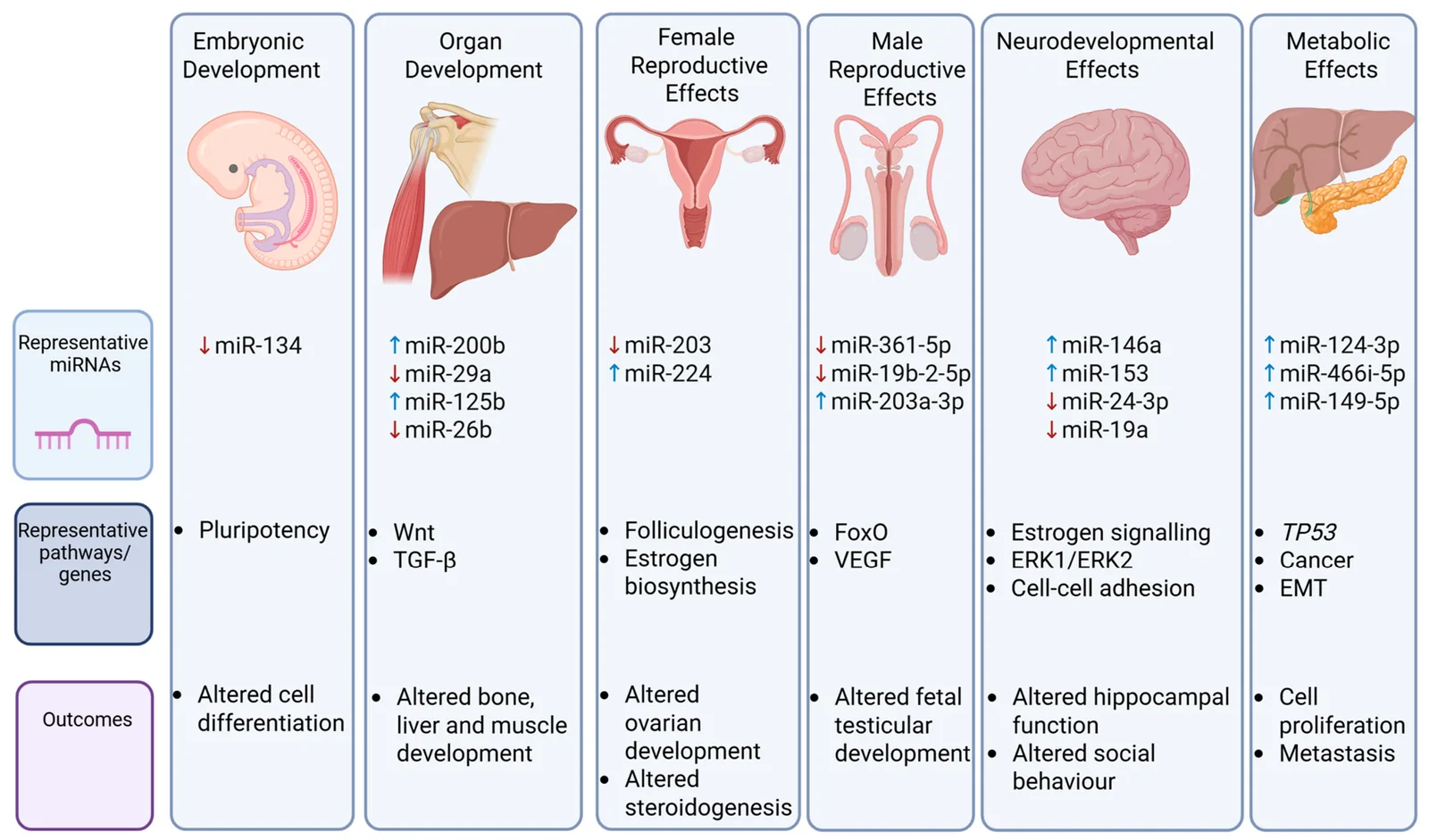
Summary of BPA-induced miRNA dysregulation following developmental exposure. Red arrow, downregulated; blue arrow, upregulated; miR, microRNA; Wnt, wingless + integration-1; TGF-β, transforming growth factor beta; FoxO, forkhead box O; VEGF, vascular endothelial growth factor; ERK, extracellular signal regulator kinase; *TP53*, tumor protein p53; EMT, epithelial–mesenchymal transition. The illustration was created on the 15 July 2026 using BioRender.com (with publication license).

Figure 5 provides a summary of transgenerational effects of BPA-induced miRNA dysregulation.

## 8. Other Health Effects of BPA

BPA exposure has also been shown to disrupt immune function. In a study by Liu et al. [93], Carp fish spleen lymphocytes were exposed to BPA (1 nM, 5 nM, 10 nM), resulting in decreased expression of miR-27b-3p, which coincided with an increase in its target gene CYP1B1 (Table 5). This dysregulation led to the accumulation of reactive oxygen species (ROS) and impairment of antioxidant enzymes such as SOD, GSH, and CAT, triggering oxidative stress and activating the mitochondrial apoptotic pathway. As a result, the expression of pro-apoptotic genes, including *BAX*, *CytC*, *Caspase-9*, and *Caspase-3*, was increased, while anti-apoptotic *BCL-2* was downregulated. The apoptotic effects of BPA exposure were partially reversed by overexpressing miR-27b-3p in transfection experiments, suggesting that miR-27b-3p may play a protective role against oxidative stress-induced apoptosis. Target prediction tools, such as TargetScan, indicated that miR-27b-3p may also target CYP1B1 in humans, highlighting the potential for BPA-induced immune dysfunction through miRNA-mediated regulation.

In bone marrow mesenchymal stem cells (BMSCs), BPA upregulated CD36, impairing autophagy by sequestering ATG9a at the Golgi, reducing osteogenic markers (ALP and RUNX-2), and disrupting autophagic flux (elevated LC3B-II/I conversion, impaired p62 degradation) [94]. Exosomes from BPA-treated BMSCs contained elevated levels of miR-148a-3p, miR-214-3p, and miR-146a-5p, which, when internalized by RAW264.7 macrophages, increased expression of mRNA osteoclastogenic markers *TRAP*, *c-Fos*, and *NFATC1*. CD36 knockdown restored autophagy, normalized miRNA levels, and reduced osteoclastogenic potential. In a male SD rat mandibular defect model, the authors also showed that BPA (oral gavage, 6 weeks, 10 μg/kg/3 days) impaired bone repair in a CD36-dependent manner, an effect reversed by CD36 silencing, but no miRNA or mRNA analysis was performed in vivo.

## 9. Discussion

This narrative review synthesizes current evidence on BPA-induced miRNA dysregulation across human, in vivo, and in vitro studies, highlighting its profound effects on metabolic, reproductive, oncogenic, and developmental pathways. The findings underscore BPA’s role as an epigenetic disruptor, with miRNA alterations contributing to both short-term and generational health effects, often in a sex-dependent manner. The existing literature exhibits considerable heterogeneity in study design, tissue samples, miRNA profiling methods, and BPA exposure doses, with a limited number of human studies.

The studies reviewed utilized a broad range of BPA exposure concentrations. Notably, low concentrations in the nanomolar to micromolar range (e.g., 10 nM in human NPC CNE2 cells [46], 20 nM in human granulosa cells [60], and 0.02–2 μM in human HepG2 cells [95]) were sufficient to induce significant alterations in miRNA expression, as reported in approximately 15 studies (Table 1 and Table 2). These low-dose effects are concerning as they fall within or below concentrations detected in human biomonitoring studies (0.1–10 ng/mL in serum and 50 ng/mL in urine) [1,10] and are substantially lower than the current tolerable daily intake (TDI) of 0.2 ng/kg body weight/day [12]. This suggests that current regulatory limits may not adequately protect against BPA’s epigenetic effects. Similarly, higher concentrations such as 100 μM in hypothalamic neurons [30], 2000–20,000 ng/mL in granulosa cells [60], and 1–5 mM in carp fish lymphocytes [93] demonstrated pronounced effects, though these exceed typical human exposure levels by several orders of magnitude. Circulating free BPA concentrations in humans are typically reported in the low nanomolar range (approximately 0.5–5 nM). Therefore, while high-dose studies provide mechanistic insights, their direct translational relevance to human health is limited to specific occupational or extreme exposure scenarios. Several studies also highlighted nonmonotonic dose–response relationships. For instance, miR-708-5p was upregulated at 1 h but downregulated after 24 h with 100 μM BPA in hypothalamic neurons [31], while miR-125b in granulosa cells exhibited downregulation at 200 ng/mL but upregulation at 20,000 ng/mL [60]. Similarly, miR-337-3p in human preadipocytes showed upregulation at 10 μM but no change at 10 nM [28]. In pancreatic islets, miR-338 was downregulated at high BPA doses while miR-200a was downregulated at low doses [27]. These nonmonotonic dose–response relationships, a recognized characteristic of EDCs, highlight the complexity of BPA-induced miRNA regulation, whereby biological responses do not necessarily follow a linear relationship with dose, posing challenges for conventional toxicological risk assessment.

Overall, miRNA upregulation was more frequently observed than downregulation, with examples including miR-21 across multiple cancer types, including breast [47], ovarian [54], and prostate [52] tissue, and miR-214-3p in male reproductive cells [72,73]. The miR-200 family showed context-dependent regulation in ovarian cancer cells; miR-200c was consistently upregulated, whereas miR-200a/b varied by cell line [55]. Conversely, other miRNAs were predominantly downregulated, such as miR-26b [47,51] in breast cancer cells and sheep liver and skeletal muscle [82], though miR-26b showed upregulation in placenta [67,68]. Tissue-specific responses were also evident for miR-146a-5p, which was upregulated in Leydig cells [75] and colon cancer cells [44] but downregulated in hypothalamic neurons [88]. Similarly, miR-338 was downregulated in pancreatic islets [27] but upregulated in liver tissue [29].

Since miRNAs generally act as suppressors of gene expression by binding to and inhibiting their target mRNAs, their upregulation would be expected to intensify these inhibitory effects [96]. Conversely, downregulation of miRNAs would be expected to relieve suppression of their target mRNAs, leading to increased expression of those target genes. This may result in pathological overexpression of oncogenes (e.g., downregulation of miR-15a/miR-16 in prostate cancer [52] potentially increases anti-apoptotic *BCL2*), enhanced inflammation (e.g., downregulation of miR-181b in endometrial cells [66] may upregulate *VEGFB/VEGFC*, promoting aberrant angiogenesis), or metabolic dysregulation (e.g., downregulation of miR-122 in the liver [33] may relieve suppression of lipogenic genes, contributing to NAFLD). Other downregulated miRNAs include miR-125b and miR-34c, which were reduced in ovarian dysfunction and spermatogenesis, respectively, affecting estrogen and p53 signaling [60,71].

Of the 50 studies reviewed, 25 employed in vitro models (primarily human cancer cell lines), 18 used in vivo animal models (predominantly Sprague–Dawley rats and C57BL/6 mice), and 7 were human epidemiological or ex vivo human tissue studies. Quantitative real-time PCR (qRT-PCR), recognized as the gold standard for miRNA detection due to its superior sensitivity and specificity [97], was used in 40 studies. Twelve employed microarrays, which have limited sensitivity and sequence discrimination [98], while only four used next-generation sequencing, which remains underutilized in BPA research [99]. This methodological heterogeneity underscores the critical need for standardization to facilitate meaningful cross-study comparisons.

The let-7 family (let-7a, b, c, f, g and their isoforms) emerged as the most frequently dysregulated miRNA family following BPA exposure, with at least 16 reported instances of altered expression across 12 or more studies. The direction of let-7 family dysregulation was inconsistent across studies, with reports of both upregulation and downregulation depending on tissue type, exposure duration, and disease context. BPA exposure induced upregulation of let-7b in mouse Sertoli TM4 cells [71], let-7a-2-3p in human placenta [68], and let-7g-5p in granulosa cells [60], while let-7c was downregulated in endometrial stromal cells [66]. Dysregulation of let-7a-5p in endometrial carcinoma cells [53]; let-7f, let-7c, and let-7g in breast cancer cells [47]; and let-7c-2-3p and let-7a-1-3p in rodent pancreatic cells [29] was also reported. Key pathways associated with the let-7 family included estrogen signaling, steroidogenesis, and placental dysfunction. Despite inconsistent patterns of regulation, common associated pathways suggest that dysregulation of the let-7 family due to BPA exposure may impact endocrine and reproductive regulation.

The miR-200 family (miR-200a,b,c, miR-141, miR-429) was the second most frequently dysregulated miRNA family, with at least 10 reported instances of altered expression across eight or more studies, though expression patterns were context-dependent. miR-200a and b showed both up- and downregulation in ovarian cancer cells, with downregulation associated with ERα signaling and EMT [55], while miR-200a, b, and c were downregulated in rat penile tissue and associated with inflammation, EMT, and fibrosis [74] and miR-200 was upregulated in sheep fetal liver [82]. Upregulation of miR-200c/141 promoted metastasis via PTEN targeting in colon cancer [45]. The miR-200 family was strongly associated with BPA-induced EMT and estrogen signaling in both gynecological and colon cancers [45,55].

miR-21 was the most frequently reported upregulated miRNA following BPA exposure, with upregulation observed in at least 13 studies. However, these studies encompass diverse biological systems and should be interpreted as an overall trend rather than a universal response. Upregulation was reported in placental models [67], bovine granulosa cells [62,63], bovine cumulus–oocyte complexes and embryos [61,63,64,65], and human placental samples from BPA-polluted areas [68]. Contrastingly, no difference in expression was observed in BPA-treated sperm blastocytes [78]. Given the established role of miR-21 in regulation of oocyte maturation, its consistent regulation across these studies suggests that it is a key mediator of BPA-induced reproductive toxicity. miR-21 was also upregulated in ovarian [54], breast [47], liver [40], and colon cancer cells, as well as in lung fibroblasts [44], and appears to be strongly implicated in cancer progression via SERPINB5, PDCD4, MAPK, and p38 pathways [26,47]. In metabolic tissue, BPA exposure upregulated miR-21 in the pancreatic islets of mice [27], although a contrasting study in murine preadipocytes reported downregulation of miR-21a-5p, suggesting tissue-specific regulatory differences [26].

The miR-29 family (miR-29a, miR-29b) was also commonly dysregulated, with at least nine reported instances of altered expression across six studies. Prenatal BPA exposure downregulated miR-29a in sheep fetal liver and muscle [82], but upregulated it in bovine oocytes and embryos [61] and downregulated it in bovine arrested 8-cell embryos [65]. In male reproductive models, BPA downregulated miR-29a-5p in mouse GC-2 cells [72] and upregulated miR-29b-3p in human sperm [77]. In human placenta from BPA-polluted areas, miR-29 was upregulated and linked to p53, ErbB, Toll, mTOR, and disease pathways [68].

The miR-26 family (primarily miR-26a, miR-26a-5p, and miR-26b) was dysregulated in at least nine instances across eight studies, being predominantly downregulated. BPA downregulated miR-26b in MCF-7 and MCF-7F cells, promoting proliferation via Rab31 [47,51], sheep fetal muscle [82], and miR-26a-5p in rat pancreatic islets [29]. BPA also downregulated miR-26a-5p in e-hPDLSCs, suggesting a role in angiogenesis [37]. In contrast, two placental studies reported miR-26b upregulation [67,68], indicating tissue-specific differences.

Finally, miR-146a, with seven reported dysregulations across six studies, was consistently upregulated across multiple BPA exposure models, playing key roles in NF-κB-mediated inflammation and immune regulation in reproductive [75] and neurodevelopmental [89] processes. BPA upregulated miR-146a in placental cell lines (3A, HTR-8), reducing proliferation [67], and human placental samples, linking to cancer and endocrine pathways [68]. In Leydig cells, miR-146a-5p upregulation impaired steroidogenesis via Mta3, decreasing testosterone [75]. In colon cancer cells and lung fibroblasts, BPA elevated miR-146a-5p, associated with TGFB1, MYC, ErbB, BRCA1, and EGFR pathways [44]. In the offspring hippocampus, maternal BPA upregulated miR-146a, correlating with social behavior and body fat [89]. Elevated levels of miR-146a-5p were also reported in BMSCs following BPA exposure, associating with impaired bone remodeling via a CD36-dependent mechanism [94]. Conversely, no difference in expression was observed in bovine COCs, arrested 8-cell embryos, or blastocysts with 0.05 mg/mL BPA exposure [65].

These collective findings across the let-7 family, miR-200 family, miR-21, miR-29 family, miR-26 family, and miR-146a underscore that BPA’s epigenetic impact is broad and involves multiple miRNAs, with directionality and functional outcomes being highly dependent on the specific tissue, cell type, and disease model. Beyond the most frequently cited families, several other miRNAs were recurrently dysregulated following BPA exposure. These included miR-19 and miR-34, with six reported dysregulations across 5 studies; miR-125, miR-181, miR-203, and miR-27, with five dysregulations each across 4–5 studies; and miR-155, miR-199, miR-221, miR-222, miR-335, and miR-499, with four dysregulations across 4 studies. Key disrupted pathways included PI3K/AKT/mTOR, MAPK, NF-κB, estrogen signaling, and Wnt/β-catenin. miR-21, miR-200, and miR-19a were linked to cancers, miR-338 and miR-122 to metabolic disturbances, and miR-146a and miR-153 to neurodevelopmental dysfunction. Sex- and generation-specific effects were also evident across a number of studies. Prenatal BPA in male rats downregulated hippocampal miR-24-3p (altering interneuron function) and testicular miR-361-5p/miR-19b-25p (disrupting FoxO/VEGF pathways), while females showed upregulated miR-153 (metabolic) [86,87,88]. BPA upregulated Leydig cell miR-146a-5p (impairing steroidogenesis) [75], while in placental cells, miR-146a upregulation reduced proliferation [67] and granulosa cell alteration of miR-224 increased aromatase/estrogen activity (linking to PCOS) [85]. In animal studies, prenatal sheep exposure altered fetal miR-200b and miR-29a (liver/muscle) [82] and rat maternal exposure disrupted oocyte miR-34c/miR-10b [61]. These findings highlight the need for further research into miRNA-based biomarkers and therapeutic targets, particularly given potential heritable dysregulation.

Despite consolidating current evidence on BPA-miRNA interactions, this review acknowledges several key limitations. Heterogeneity in BPA doses, experimental models, and miRNA methodologies and the lack of standardized reporting of exposure validation and miRNA extraction protocols hinder direct comparison between studies. Human data are largely correlative, not causal; disease states themselves alter miRNA profiles, so observed changes may be epiphenomena rather than direct BPA effects. A major gap also lies in the scarcity of human data; most of the available research is based on in vitro or animal models, underscoring the need for more human biomonitoring studies. Furthermore, none of the epidemiological studies included in this review adjusted for potential co-exposure to other EDCs. Real-world exposure scenarios often involve simultaneous exposure to multiple EDCs, which may potentiate epigenetic effects. For example, co-exposure to BPA and phthalates synergistically altered miR-27b-3p expression in carp lymphocytes, exacerbating oxidative stress [93]. Whether the observed miRNA alterations can be attributed exclusively to BPA exposure remains uncertain, as co-exposure to other EDCs may independently or synergistically influence miRNA expression. In vitro and animal studies may not fully replicate human biology or translate directly. Many studies also lacked functional validation (e.g., miRNA knockdown/overexpression), and while circulating miRNAs are commonly used as biomarkers, they may not accurately represent tissue-specific changes. Therefore, associations should be interpreted as suggestive rather than definitive. While this study focused on BPA, BPA analogs (e.g., BPS, BPF) may similarly dysregulate miRNAs, such as is reported by the BPS and BPF [44]. Finally, the review relied on published data, which may introduce publication bias. Relatively few included studies reported unchanged miRNA expression following BPA exposure, potentially indicating that null findings are underreported in the literature. Additionally, many studies focused on those miRNAs showing statistically significant changes, potentially introducing selective reporting bias. Consequently, the predominance and consistency of certain expression patterns may be overestimated. In addition, restricting the search to English language studies may have excluded relevant research.

## 10. Future Directions

Despite growing evidence linking bisphenol A exposure with altered miRNA expression and disease pathogenesis, several important knowledge gaps remain and there is a need to establish causality, identify biomarkers, and define clinically meaningful exposure thresholds. Future research should move beyond descriptive associations toward integrated translational approaches capable of establishing causality and identifying biomarkers. For example, more research on extracellular vesicle-associated miRNAs is needed. From a clinical perspective, there remains a need to determine whether altered miRNA expression represents merely a biomarker of exposure or a true mechanistic driver of disease.

There is a need to define clinically meaningful exposure thresholds: EFSA recently reduced the tolerable daily intake for BPA to 0.2 ng/kg body weight/day, approximately 20,000-fold lower than the previous temporary value; however, this has been criticized [100].

One of the most important priorities is the development of large, prospective, longitudinal, human cohort studies incorporating repeated BPA exposure measurements alongside serial miRNA profiling. Given the short biological half-life of BPA and substantial intra-individual variability in exposure, reliance on single urine or serum measurements may underestimate chronic exposure and obscure biologically relevant effects. These studies should prioritize miRNAs that were most consistently dysregulated across multiple studies and biological systems. The current evidence suggests that the let-7 family, miR-21, and the miR-200 family are particularly promising candidates because of their recurrent dysregulation across multiple studies and their roles in endocrine signaling, carcinogenesis, and reproductive function. The miR-29 and miR-26 families, along with miR-146a and metabolically relevant miRNAs such as miR-122 and miR-338, also warrant further investigation as candidate biomarkers of BPA-associated disease.

Future studies should also increasingly focus on environmentally relevant low-dose exposure levels. Many current experimental studies utilize BPA concentrations substantially higher than those encountered under typical human exposure conditions, limiting translational interpretation. Future studies should address real-world chemical mixtures rather than isolated BPA exposure.

Another major area for future investigation is the integration of multi-omics technologies. Combining miRNA profiling with transcriptomics, proteomics, metabolomics, epigenomics, and exposomics may help identify molecular networks disrupted by BPA exposure. Systems biology approaches could clarify how BPA-induced miRNA dysregulation interacts with inflammatory signaling, oxidative stress, mitochondrial dysfunction, steroidogenesis, insulin signaling, and developmental programming pathways. Integration with network analysis and machine learning may further identify central regulatory hubs and disease-specific miRNA signatures.

The growing concern surrounding BPA toxicity has led to the use of a number of structurally similar analogs, including BP-AF, BP-AP, BPB, BPF, BPS, and BPZ. Although introduced as safer alternatives, increasing evidence suggests that they possess similar, if not greater, endocrine-disrupting activity than BPA, as well as synergistic and additive toxic effects [101]. With the progressive replacement of BPA and the increasing production of and human exposure to these analogs, there is a critical need to profile miRNA dysregulation induced by these replacement bisphenols. Such studies will help elucidate whether BPA analogs share common molecular mechanisms with BPA or induce distinct patterns of miRNA dysregulation, thereby informing risk assessment and regulatory decision-making.

## 11. Conclusions

The evidence reviewed here demonstrates that BPA exposure disrupts miRNA expression, influencing key pathways involved in metabolic disorders, cancer, reproductive dysfunction, and developmental abnormalities. Analysis of the included studies reveals let-7, miR-21, and miR-200 as the most frequently cited miRNA families, with miR-21 predominantly upregulated across reproductive and oncogenic models, whereas let-7 and miR-200 displayed tissue- and exposure-dependent regulation. Similarly, the miR-146, miR-29, and miR-26 families were frequently dysregulated, with miR-146a consistently upregulated across placental and cancer models, while miR-29 and miR-26 were predominantly downregulated. Collectively, these findings highlight that BPA’s epigenetic effects vary by tissue, species, and experimental model, underscoring the complexity of BPA’s epigenetic influence. These findings emphasize the need for standardized methodologies, longitudinal human studies, and validation of non-invasive miRNA biomarkers to establish causal links between BPA exposure and disease. Advancing these efforts will be critical for informing evidence-based policies and targeted therapies to mitigate BPA-associated health risks.

## Figures and Tables

**Table 3:** BPA-induced miRNA dysregulation and female reproductive effects.

BPA Level/Dose	Study Design; Sample (*n*)	miRNA	miRNA Expression	Methodology	miRNA Target Analysis	Signaling Pathways; Target Genes	Ref.
48 h × 20, 200, 2000, 20,000 ng/mL	In vitro; human granulosa cells (*n* = 5–10)	Let-7g-5p	↑	OpenArray, qRT-PCR	Bioinformatic analysis, IPA, qRT-PCR	Follicular development, oocyte maturation, and human reproduction; *AKAP8*, *ATP6V1F*, *FADD*, *IGF1*, *MECP2*, *PPARG*	[60]
		miR-191-5p	↑				
		miR-532	↑				
		miR-125b					
		miR-212-3p	↓				
		miR-324-5p	↓				
		miR-27b-3p	↓				
		miR-335	↓				
		miR-572	↓				
24 h × 0.05 mg/mL	In vitro; matured bovine cumulus–oocyte complexes (COCs), oocytes, cumulus cells and embryos (*n* = 3)	miR-21	↑	qRT-PCR	N/P	Oocyte maturation;	[61]
		miR-155	↑				
		miR-29a	↑				
		miR-34c	↓				
		miR-10b	↓				
12 h × 0.05 mg/mL	In vitro; bovine granulosa cells (*n* = 3)	miR-21	↑	qRT-PCR	qRT-PCR, Western blotting	*PDCD4*, *PTEN*, *VMP1*, *STAT3*,	[62]
		miR-10b	↓				
		miR-155	↑				
Last 12 h of 24 h maturation × 0.05 mg/mL	In vitro; bovine cumulus–oocyte complexes (COCs), granulosa cells (GCs) (*n* = 3)	miR-21	↑	anti-miR-21 LNA knockdown, qRT-PCR	qRT-PCR, Western blotting	DNA methylation; *DNMT1*, *DNMT3A*, *DNMT3B*, *TET1*, *TET2*, *TET3*, *TDG*	[63]
Last 12 h of 24 h maturation × 0.05 mg/mL	In vitro; bovine cumulus–oocyte complexes (COCs), denuded oocytes, cumulus cells (*n* = 3)	miR-21	↑	anti-miR-21 LNA, qRT-PCR	qRT-PCR	PTEN/PI3K/Akt; *Cx37*, *Cx43*, *Cx26*	[64]
		miR-378a	↑				
		miR-96	↓				
		miR-130a	↑				
		miR-155	↑				
Last 12 h of 24 h maturation × 0.05 mg/mL	In vitro; bovine cumulus–oocyte complexes (COCs), arrested 8-cell embryos, blastocysts (*n* = 3)	miR-21	↑	anti-miR-21 LNA knockdown, qRT-PCR	N/P	N/P	[65]
		miR-34c	↓↑				
		miR-155	↓				
		miR-224	↓				
		miR-103a					
		miR-130a	↓↑				
		miR-499	↓				
		miR-10b	↓↑				
		miR-29a	↓				
		miR-146a					
		miR-378	↑				
4, 8, 16, 24 h × 3 μM	In vitro; human endometrial stromal cells (*n* = 3)	miR-181b	↓	qRT-PCR	qRT-PCR, TargetScan, ELISA	*VEGFB*, *VEGFC*	[66]
		miR-27b	↓				
		Let-7c	↓				
6 days × 25.0 ng/µL	In vitro; human SV40 transformed placental cell lines (3A, and HTR-8) (*n* = 3)	3A and HTR-8		Microarray, qRT-PCR	Literature review	Cancer, inflammatory response, and cell growth;	[67]
		hsa-let-7f	↑				
		hsa-let-7g	↑				
		hsa-miR-146a	↑				
		hsa-miR-21	↑				
		HTR-8					
		hsa-let-7i	↑				
		hsa-miR-106a	↑				
		hsa-miR-106b	↑				
		hsa-miR-155	↑				
		hsa-miR-16	↑				
		hsa-miR-19b	↑				
		hsa-miR-20a	↑				
		hsa-miR-26b	↑				
		hsa-miR-29a	↑				
		hsa-miR-335	↑				
		hsa-miR-376c	↑				
Approx. mean 120 ng/g	Case–control study; placenta samples (*n* = 40 (polluted with therapeutic abortion), 40 (controls), *n* = 3)	miR-1243	↑	Microarray, qRT-PCR	Bioinformatic analysis, miRanda, TargetScan, KEGG	Neural disease; *IRAK1*, *MYT1*, *ROBO1*, *LRRTM2*, *GRID1*, *SORT1*, *BCL11A*, *SYT1*, *NPAS4*, *MLL2*, *DNAL1*, *EDNRB* endocrine system; *TP53* and cardiovascular disease; *ABL2*, *EDNRB* cancer; *ErbB*, *p53*, *Toll*, *mTOR*	[68]
		miR-519e-3p	↑				
		miR-371-3p	↑				
		miRplus-c1066	↑				
		miR-146a	↑				
		miR-29a	↑				
		miR-1256	↑				
		miR-21	↑				
		miR-26b	↑				
		miR-29a	↑				
		miR-335	↑				
		miR-376c	↑				
		miR-605	↓				
		miR-571	↓				
		miR-23b-5p	↓				
		miR-885-5p	↓				
		miR-1471	↓				
		let-7a-2-3p	↓				
264.9 pg/g	Cohort study; human placental tissue (*n* = 63)	N/S	N/S	miRNA profiling	N/P	N/S	[69]

(N/S), not specified; ↑, upregulation; ↓, downregulation; (N/P), not performed.

**Table 4:** BPA-induced miRNA dysregulation and male reproductive effects.

BPA Level/Dose	Study Design; Sample (*n*)	miRNA	miRNA Expression	Methodology	miRNA Target Analysis	Signaling Pathways; Target Genes	Ref.
3, 24 h × 20 μg/mL	In vitro; mouse SC line (TM4) (*n* = 3)	miR-181d miR-296-5p miR-466a-5p miR-let-7b miR-324-5p miR-106b miR-705 miR-103 miR-10b miR-34c miR-221 miR-463 miR-669a miR-466b-5p miR-574-5p miR-93 miR-690 miR-222 miR-33 miR-451 miR-23b miR-468 miR-466c-5p miR-500 miR-151-5p miR-199b miR-99b miR-378 miR-467a miR-26a miR-24 miR-467e miR-199a-5p miR-22 miR-31 miR-199a-3p miR-27a	↑ ↑ ↑ ↑ ↓ ↓ ↓ ↓ ↓ ↓ ↓ ↓ ↓ ↓ ↓ ↓ ↓ ↓ ↓ ↓ ↓ ↓ ↓ ↓ ↓ ↓ ↓ ↓ ↓ ↓ ↓ ↓ ↓ ↓ ↓ ↓ ↓	Microarray	Microarray, bioinformatic analysis, GO, KEGG, GeneSpring, JAK	Genes associated with cell cycle/reproduction and metabolism	[71]
48 h × 120 μM	In vitro; GC-2 cells (a murine spermatocyte-like cell line) (*n* = 3)	miR-214-3p miR-335-5p miR-29a-5p miR-152-5p miR-340-3p	↓ ↓ ↓ ↓ ↓	Sequencing, qRT-PCR	Sequencing, bioinformatic analysis, DIANA, GO, KEGG	*Clock*, *Clasp1*, *Fbxl7*, *Htt*, *Qki*	[72]
48 h × 120 μM	In vitro; GC-2 germ cells (a mouse spermatocyte-like cell line) (*n* = 3)	miR-214-3p	↓	qRT-PCR	qRT-PCR, Western blot analysis, luciferase reporter assay, bioinformatics analysis, miRanda, DIANA	PI3K-mTOR; *Akt1*, *Cd59a*, *Csnk1e*, *Pkn3*, *Sdf2l1*	[73]
4.5 months × 0.1, 1 mg/kg/day	Male Fischer 344 rats; penile tissue (*n* = 3)	miR-568 miR-451-5p miR-664-1-5p miR-296-3p miR-377-3p miR-1224 miR-665 miR-182 miR-672-5p miR-483-5p miR-1306-3p miR-210-3p miR-3584 miR-494-3p miR-206-3p miR-200c miR-328a miR-347 miR-200b miR-6216 miR-6215 miR-429 miR-200a miR-205 miR-203a	↑ ↑ ↓ ↓ ↓ ↓ ↓ ↓ ↓ ↓ ↓ ↓ ↓ ↓ ↓ ↓ ↓ ↓ ↓ ↓ ↓ ↓ ↓ ↓ ↓	Microarray	DNA microarray, literature review	Inflammation and EMT; *ERRFI1*, *E-NCAM*, *KRT 1*, *4*, *7*, *8*, *14*, *15*, *19*, *CDH1; CXCL1*, *IL6*, *IL1B*, *CCL2*, *PLAU*, *COX2*, *PTGS2*, *CD248*, *MAP3K8*, *CADM3*, *NOS2*	[74]
70 days × 0.5, 1.5 4.5 mg/kg/day 48 h × 50 nM	Male C57B/6 mice; murine testis (germ cells (GCs), SCs, and LCs) In vitro; MA-10 cells (*n* = 3)	miR-146a-5p	↑	qRT-PCR, chromogenic in situ hybridization	qRT-PCR, bioinformatic analysis, TargetScan, Mirna, immunohistochemistry, immunoblotting, luciferase reporter assay	*Mta3*;	[75]
Approx. 3 nmol/L	Case–control study; seminal plasma samples (*n* = 20 (azoospermia), 46 (oligoasthenoteratozoospermia), 50 (controls)	miR-let-7a miR-let-7b miR-let-7c miR-518f	↑ ↑ ↑ ↓	qRT-PCR	Literature review	*Cyp19*	[76]
0.175 ng/mL ± 0.133 ng/mL	Cross-sectional study; 102 Portuguese male donors. Seminal plasma, sperm (*n* = 15 for sequencing)	miR-451a miR-6124 miR-148b-5p miR-6832-3p miR-1271-5p miR-29b-3p miR-29c-3p miR-486-3p miR-4661-5p miR-499b-5p miR-4423-5p miR-185-5p miR-329-3p miR-34b-3p miR-132-3p	↑ ↑ ↑ ↑ ↑ ↑ ↑ ↑ ↓ ↓ ↓ ↓ ↓ ↓ ↓	TargetScan and miRDB	Bioinformatics	PI3K-Akt, MAPK, TGF-β, cAMP;	[77]
4 h × 0.05 mg/mL	In vitro; bovine sperm and BPA-treated sperm blastocysts (*n* = 3)	miR-191 miR-30a miR-100 miR-34c miR-21 miR-33b miR-324 miR-130a	↑	qRT-PCR	N/P	N/S	[78]

(N/S), not specified; ↑, upregulation; ↓, downregulation; (N/P), not performed.

**Table 5:** BPA-induced miRNA dysregulation and developmental programming and other health effects.

BPA Level/Dose	Study Design; Sample (*n*)	miRNA	miRNA Expression	Methodology	miRNA Target Analysis	Signaling Pathways; Target Genes	Ref.
24 h (mESC) × 0.04, 1, 25, 100 μM 2, 4, 6 days (mEB) × 0.04, 1, 25, 100 μM	In vitro; mESC and mEB (*n* = 3)	miR-134	↓	qRT-PCR	qRT-PCR, Western blotting	*Oct4*, *Sox2*, *Nanog*, *Gata4*, *Sox17*, *Sma*, *Desmin*, *Nestin*, *Fgf5*	[81]
60 days × 0.5 mg/kg/day	Female Suffolk sheep; liver and skeletal muscle (*n* = 3)	Liver miR-200b miR-30b miR-409 miR-26b miR-125b miR-543 miR-154a miR-25 miR-22 miR-191 miR-381 miR-136 miR-382 miR-29a miR-323a Muscle miR-26b miR-29a miR-181a-1 miR-125b miR-127 miR-541	↑ ↓ ↑ ↓ ↑ ↑ ↓ ↑ ↑ ↑ ↓ ↓ ↑ ↓ ↑ ↓ ↓ ↑ ↑ ↑ ↑	Sequencing	Bioinformatic analysis, Biocarta, EMHN metabolic, GO, KEGG, Panther, transcription factor databases		[82]
14 days × 14.03 ± 0.89 μg/L	Male rare minnows (Gobiocypris rarus); sperm (*n* = 3)	PC-3p-133610_29 PC-3p-18101_257 ccr-miR-727–3p PC-3p-2927_1428 dre-miR-31 oni-miR-7550 ssa-miR-16b-5p	N/S N/S N/S N/S N/S N/S N/S	MicroRNA sequencing, qRT-PCR	qRT-PCR, bioinformatic analysis, TargetScan, GO, KEGG	Wnt, TGF-beta, oocyte division, endocytosis, cell cycle, adhesion junction, and AMPK; *Runx1*, *Runx2a*, *bmp2a*, *and bmp5*	[83]
60 days × 0.5 mg/kg/day	Gestationally exposed fetal Suffolk ewes; ovarian tissue (*n* = 5)	miR-203 +54 miRNA	↓ ↓	qRT-PCR	qRT-PCR, bioinformatic analysis, TargetScan	Gonadal differentiation, folliculogenesis, and insulin homeostasis; *Cyp19*, *5α-reductase*, *ADIPOR*, *ACVR2B*, *AR*, *AREG*, *BMPR1A*, *BMP6*, *ESR1*, *GDF10*, *IRS*, *INSIG*, *INHBB*, *INSR*, *IDE*, *IGF*, *IGF1R*, *IGFBP*, *IGF2BP*, *KITLG*, *LDLR*, *NR5A2*, *PPARa*, *PTGS1*, *PAPPA*, *PGR*, *RARB*, *RXRa*, *RARa*, *SOX*, *TGFBR*	[84]
14 days × 2.5, 250, 2500 mg/L	Gestationally exposed female Wistar rats; ovarian granulosa cells (*n* = 6)	miRNA-224	↑	qRT-PCR	Bioinformatic analysis, DAVID, Reactome, KEGG, Western blotting	Estrogen biosynthesis and ovarian steroidogenesis; *CYP19A1*	[85]
15 days × 0.05, 5 mg/kg/day	Gestationally exposed male Sprague–Dawley rat; fetal testicular tissue (*n* = 10; *n* = 3)	miR-361-5p miR-19b-2-5p miR-203a-3p	↓ ↓ ↑	Microarray, qRT-PCR	Bioinformatic analysis, miRDB, TargetScan, DAVID, GO, KEGG	FoxO and VEGF; genes involved in cancer and pancreatic secretion; genes related to RNA polymerase II promoter	[86]
50 days × 0.5, 5 mg/kg/day	Long Evans atr; dorsal hippocampus tissue (*n* = 2)	miR-24-3p	↓	RNA sequencing, qRT-PCR	RNA sequencing, qRT-PCR, GO	*Gli3*, *Pou2f1 Oct1*, *Pou2f1/Oct1*, *Pou3f2 Brn2*, *Sox6 and Sox11. Nrg1*, *Eph4*, *Fzd3*, *Tcf7L2*, *Notch2*, *Fgf14 ER-alpha*, *Pvalb*	[87]
Approx. 67days × 5, 50 mg/kg/day	Developmentally exposed adult California mice (Peromyscus californicus); hippocampal and hypothalamic tissue (*n* = 12)	miR-153 miR-181a miR-9 (female)	↑ ↓ ↑	qRT-PCR	qRT-PCR	Estrogen; *Avp*, *Esr1*, *Esr2*, *Kiss1*, *Lepr*, *Oxtr*, *Gnrh*, *Bdnf*	[88]
Approx. 67days × 5, 50 mg/kg/day	Developmentally exposed adult California mice (Peromyscus californicus); hippocampal tissue (*n* = 10)	miR-146a +87 (female) +67 (male)	↑ DE DE	RNA sequencing, qRT-PCR	qRT-PCR, bioinformatic analysis, miRror, PITA_TOP PicTar_4way, TargetRank-all, TargetScan Conserved, microCosm, miRanda Conserved, DIANA-microT, EIMMO-MirZ, miRDB, RNA22, MAMI, Map2, WEB-based Gene SeT AnaLysis, GO	Regulation of cell–cell adhesion, response to nerve growth factor, regulation of innate immune response, positive regulation of defense response, ERK1 and ERK2 cascade, and protein polyubiquitination; *Cdk5*, *Grid1*, *Klf4*, *Ptpra*, *Syt14*	[89]
5 mg/kg/day	In vivo; female and male white Sprague–Dawley rats (ages 7 to 8 weeks); hippocampal tissue	miR-19a miR-539	↓ ↓	qRT-PCR, Western blot	qRT-PCR	*GRIN2A* and *GRIN2B*, NMDA receptor-related genes	[90]
3, 6, 9, 12 weeks × 0.05 mg/kg/day 24 h × 0.1 μg/L	Gestationally exposed male ICR mice offspring; serum, adipose and liver tissue (*n* = 3) In vitro; ADSC exosome co-exposure with AML12 hepatocytes (*n* = 3)	miR-124-3p miR-466i-5p	↑ ↑	sRNA sequencing, qRT-PCR	sRNA sequencing, qRT-PCR, Western blotting, TargetScan, KEGG, miRDB, DAVID	AMPK, PPAR, Ras, IR, TNF, and MAPK; *PPARγ*, *Fgf21*, *AKT*, *Pparc*, *Fgf21*, *Slc2A4*, *Ppp1r3b*, *Hspa5*, *Nr1d1*, *Gale*	[91]
21 weeks × 50 μg/kg/day 48 h × 20 μM	Paternally exposed C57BL/6J mice; perm (F0) and liver tissue (F1) (*n* = 3–10) In-vitro; GC-2spd spermatocytes (*n* = 3)	miR149-5p miR-1a-3p miR-133a-3p miR-3068-5p miR-615-3p miR-150-5P miR-1b-5p miR-700-5p miR-486a-3p miR-145a-5p miR-871-5p miR-5119 2 × novel miR	↑ ↑ ↑ ↑ ↑ ↑ ↑ ↑ ↑ ↑ ↓ ↓ ↓	Microarray, qRT-PCR	Microarray, qRT-PCR, luciferase reporter assay, Western blotting, miRWalk, KEGG, GO, STRING, Cytoscape, OmicStudio	PI3K-Akt, insulin, IR, AMPK, NAFLD Insr–Irs1–Akt cascade, and Srebf1–Pparg–Dicer1 axis; *Lepr*, *Igfbp2*, *Srebf1*, *Pparg*, *Cyclin D1*, *Egfr*	[92]
24 h × 1, 5, 10 nM	In vitro; carp fish spleen lymphocyte cells (*n* = 3)	miR-27b-3p	↓	qRT-PCR	qRT-PCR, Western blotting, luciferase reporter assay	Mitochondrial apoptotsis; *CYP1B1*, *BAX*, *Caspase-9*, *Caspase-3*, *BCL-2*, *CytC*	[93]
N/S	In vitro; BMSC exosomes (*n* = 3)	miR-148a-3p miR-214-3p miR-146a-5p	↑ ↑ ↑	qRT-PCR	qRT-PCR, Western blotting, immunohistochemistry	Autophagy; *TRAP*, *c-Fos*, *NFATC1*	[94]

(N/S), not specified; ↑, upregulation; ↓, downregulation; (N/P), not performed.

## Data Availability

No new data were created or analyzed in this study. Data sharing is not applicable to this article.

## Abbreviations

BPA, bisphenol A (4,4′-isopropylidenediphenol); EDCs, endocrine-disrupting chemicals; miRNAs, microRNAs; mRNA, messenger RNA; EV, extracellular vesicles; GR, glucocorticoid receptor; GPR, g-protein coupled estrogen receptor; Glp1r, glucagon-like peptide 1 receptor; IPA, ingenuity pathway analysis; AgRP, agouti-related peptide; TSBs, target site blockers; Chop, C/EBP homologous protein; ER, endoplasmic reticulum; Npy, neuropeptide Y; NAFLD, non-alcoholic fatty liver disease; JNK, c-Jun N-terminal kinase; GO, gene ontology; ERK 1/2, extracellular signal-regulated kinase 1/2; MAPKAPK, mitogen-activated protein kinase-activated protein kinase; SBP, systolic blood pressure; DBP, diastolic blood pressure; NPC, nasopharyngeal carcinoma cells; HLFs, human lung fibroblasts; ERα, estrogen receptor α subtypes; *p*, phosphorylated; E2, estradiol; T, testosterone; PRL, prolactin; PCOS, polycystic ovary syndrome; IVF, in vitro fertilization; EC, endometrial cancer; EMT, epithelial-to-mesenchymal transition; LOAEL, lowest observed adverse effect level; SHBG, sex hormone-binding globulin; TM4, Sertoli testicular cells; GC-2 cells, spermatocyte-like cells; LC, Leydig cells; mESC, mouse embryonic stem cells; PAHs, polycyclic aromatic hydrocarbons; mEB, mouse embryoid bodies; cTnI, cardiac troponin I; SOX, Sry-related high-mobility-group box; FSH, follicle-stimulating hormone; VEGF, vascular endothelial growth factor; LH, luteinizing hormone; Avp, arginine vasopressin; Esr, estrogen receptor; DAVID, Database for Annotation, Visualization, and Integrated Discovery; KEGG, Kyoto Encyclopedia of Genes and Genomes; Kiss1, kKisspeptin; Lepr, leptin receptor; Gnrh, gonadotropin-releasing hormone; ROS, reactive oxygen species; MeSH, Medical Subject Headings; qRT-PCR, quantitative real-time polymerase chain reaction; PPARγ, peroxisome proliferator-activated receptor gamma; C/EBPα, CCAAT/enhancer-binding protein alpha; MAPK, mitogen-activated protein kinase; MKK3, mitogen-activated protein kinase kinase 3; GPR30, G-protein coupled estrogen receptor 30; Pdx1, pancreatic and duodenal homeobox 1; EIF2, eukaryotic initiation factor 2; MYCN, V-myc avian myelocytomatosis viral oncogene neuroblastoma derived homolog; FOXO, forkhead box O; PI3K-Akt, phosphoinositide 3-kinase-protein kinase B; KLF4, krüppel-like factor 4; Odz4, Odz, odd Oz/ten-m homolog 4; Nnat, neuronatin; SREBF1, sterol regulatory element-binding transcription factor 1; AST, aspartate aminotransferase; LDH, lactate dehydrogenase; HDL, high-density lipoprotein; Akt, protein kinase B; CASP7, caspase 7; NET1, neuroepithelial cell transforming 1; MAPK1, mitogen-activated protein kinase 1; MAPK3, mitogen-activated protein kinase 3; TGFB1, transforming growth factor beta 1; MYC, myelocytomatosis oncogene; ErbB, erythroblastic leukemia viral oncogene homolog; BRCA1, breast cancer gene 1; EGFR, epidermal growth factor receptor; CASP2, caspase 2; ESR1, estrogen receptor 1; ATR, ataxia telangiectasia and Rad3 related; BBC3, BCL2 binding component 3; MLH1, MutL homolog 1; PTEN, phosphatase and tensin homolog; RB1, retinoblastoma 1; SIRT1, sirtuin 1; STAT1, signal transducer and activator of transcription 1; TADA3, transcriptional adaptor 3; TP53PP2, tumor protein p53 pseudogene 2; CTNNB1, catenin beta 1; Wnt, wingless + integration-1; BCL2, B-cell lymphoma 2; CTSD, cathepsin D; PGR, progesterone receptor; SERPINB5, serpin family B member 5; TFF1, trefoil factor 1; JUN, Jun proto-oncogene; FAS, Fas cell surface death receptor; GABRP, gamma-aminobutyric acid type A receptor subunit pi; GSN, gelsolin; PDCD4, programmed cell death 4; PTTG1, pituitary tumor-transforming gene 1; Rab31, Ras-related protein Rab-31; KRAS, Kirsten rat sarcoma viral oncogene homolog; CDKN1A, cyclin-dependent kinase inhibitor 1A; VEGFA, vascular endothelial growth factor A; AKAP8, A-kinase anchoring protein 8; ATP6V1F, ATPase H+ transporting V1 subunit F; FADD, Fas associated via death domain; IGF1, insulin-like growth factor 1; MECP2, methyl-CpG binding protein 2; PPARG, peroxisome proliferator-activated receptor gamma; VEGFB, vascular endothelial growth factor B; VEGFC, vascular endothelial growth factor C; GREB1, growth-regulating estrogen receptor binding 1; DEPTOR, DEP domain containing MTOR interacting protein; CA12, carbonic anhydrase 12; RBBP8, RB binding protein 8; CDH1, cadherin 1; STAT3, signal transducer and activator of transcription 3; VMP1, vacuole membrane protein 1; Mta3, metastasis-associated 1 family member 3; Cyp19, cytochrome P450 family 19; OCT4, octamer-binding transcription factor 4; SOX2, Sry-related high-mobility-group box 2; NANOG, Nanog homeobox; GATA4, GATA binding protein 4; SOX17, Sry-related high-mobility-group box 17; FGF5, fibroblast growth factor 5; GLI3, GLI family zinc finger 3; POU2F1, POU class 2 homeobox 1; SOX6, Sry-related high-mobility-group box 6; SOX11, Sry-related high-mobility-group box 11; NRG1, neuregulin 1; FZD3, frizzled class receptor 3; FGF14, fibroblast growth factor 14; PVALB, parvalbumin; KISS1, kisspeptin; LEPR, leptin receptor; BDNF, brain-derived neurotrophic factor; GRIN2A, glutamate receptor ionotropic NMDA 2A; GRIN2B, glutamate receptor ionotropic NMDA 2B; CYP1B1, cytochrome P450 family 1 subfamily B member 1; SOD, superoxide dismutase; GSH, glutathione; CAT, catalase; BAX, BCL2-associated X; CytC, cytochrome C; BCL-2, B-cell lymphoma 2
